# Supplementary figures and images for: Levels of SARS-CoV-2 population exposure are considerably higher than suggested by seroprevalence surveys
Source: PLoS Comput Biol. 2021 Sep 20;17(9):e1009436. doi: 10.1371/journal.pcbi.1009436 (PMC8483393; doi:10.1371/journal.pcbi.1009436)

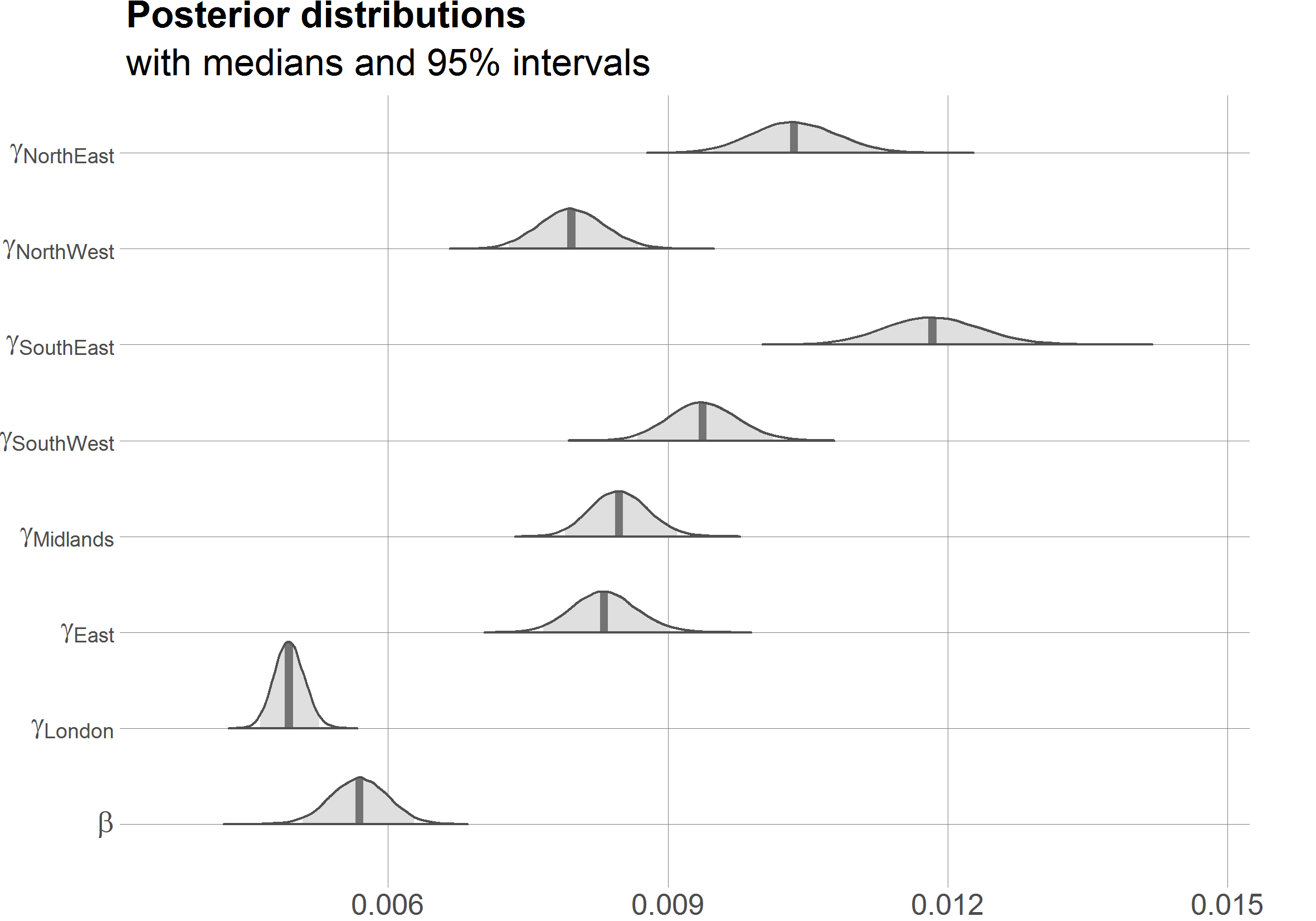

Supplement: S1 Fig — The vertical lines show the median distributions, and the grey shaded regions show the 95% CrI. (TIFF) [file pcbi.1009436.s001.tiff]

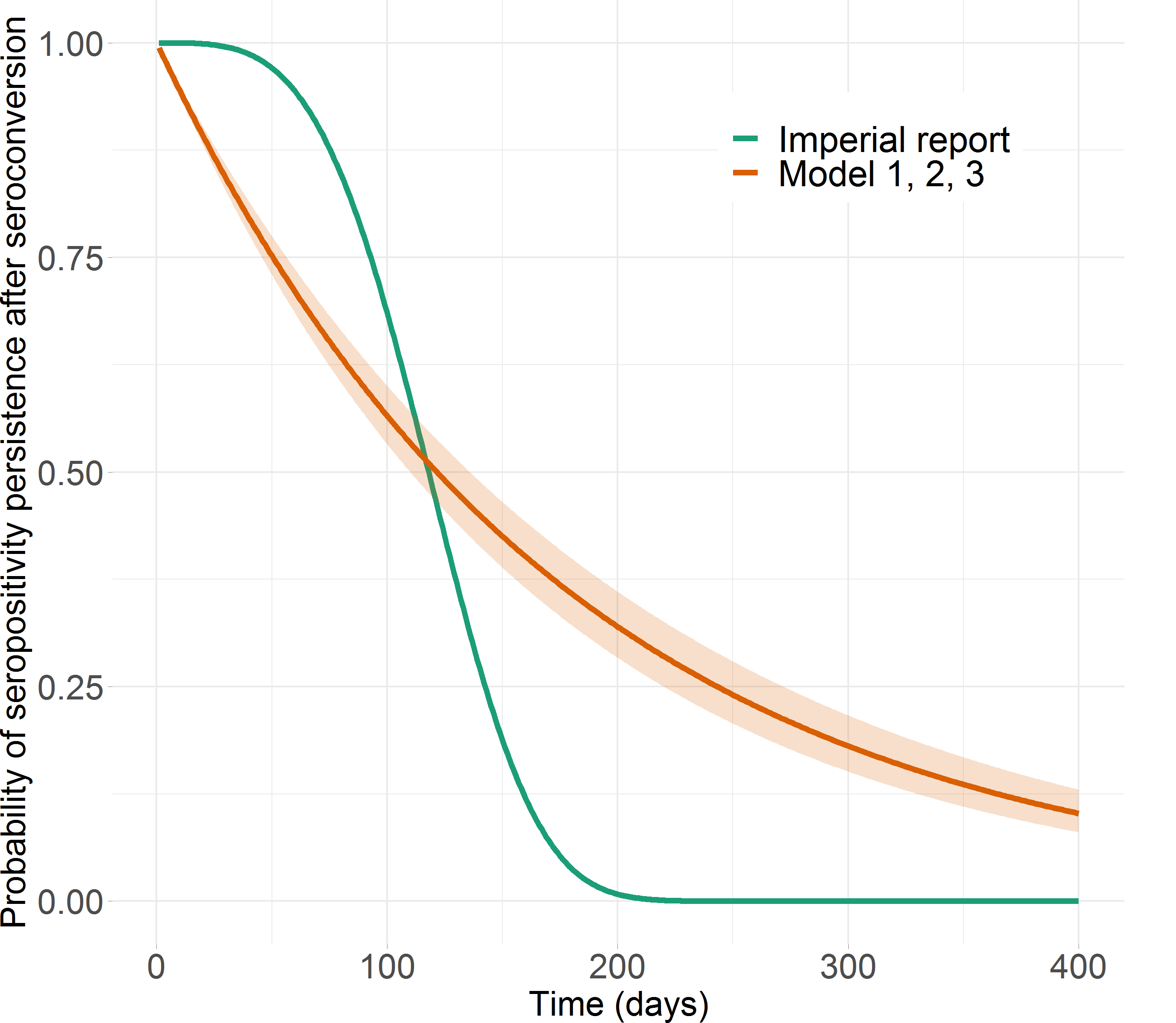

Supplement: S2 Fig — The green curve show the probability curve from [26] and the orange curve gives the median probability curve for Models 1, 2 and 3 in our study within the corresponding 95% credible intervals defined by the shaded area. See S4 Table for details on each model’s assumptions. (TIFF) [file pcbi.1009436.s002.tiff]

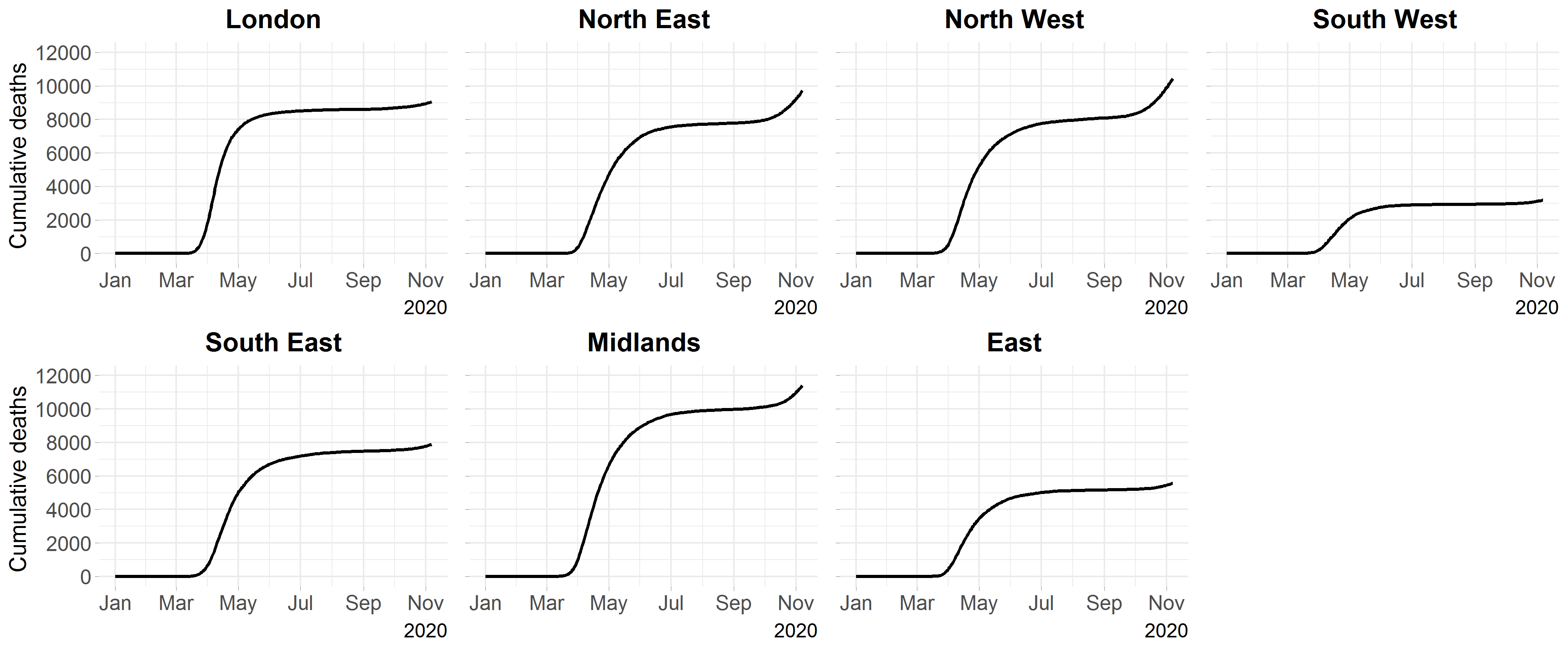

Supplement: S3 Fig — (TIFF) [file pcbi.1009436.s003.tiff]

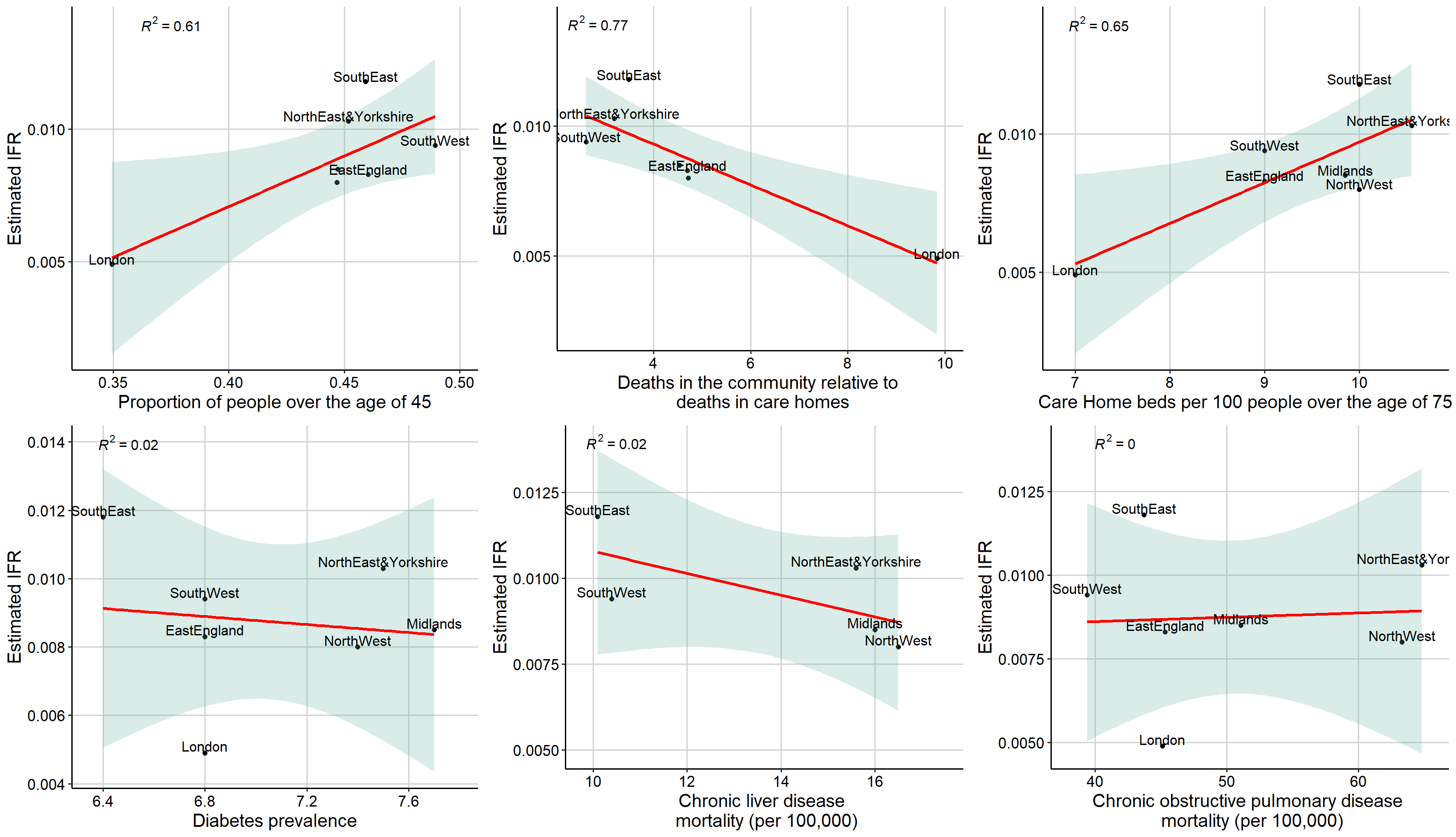

Supplement: S4 Fig — (TIFF) [file pcbi.1009436.s004.tiff]

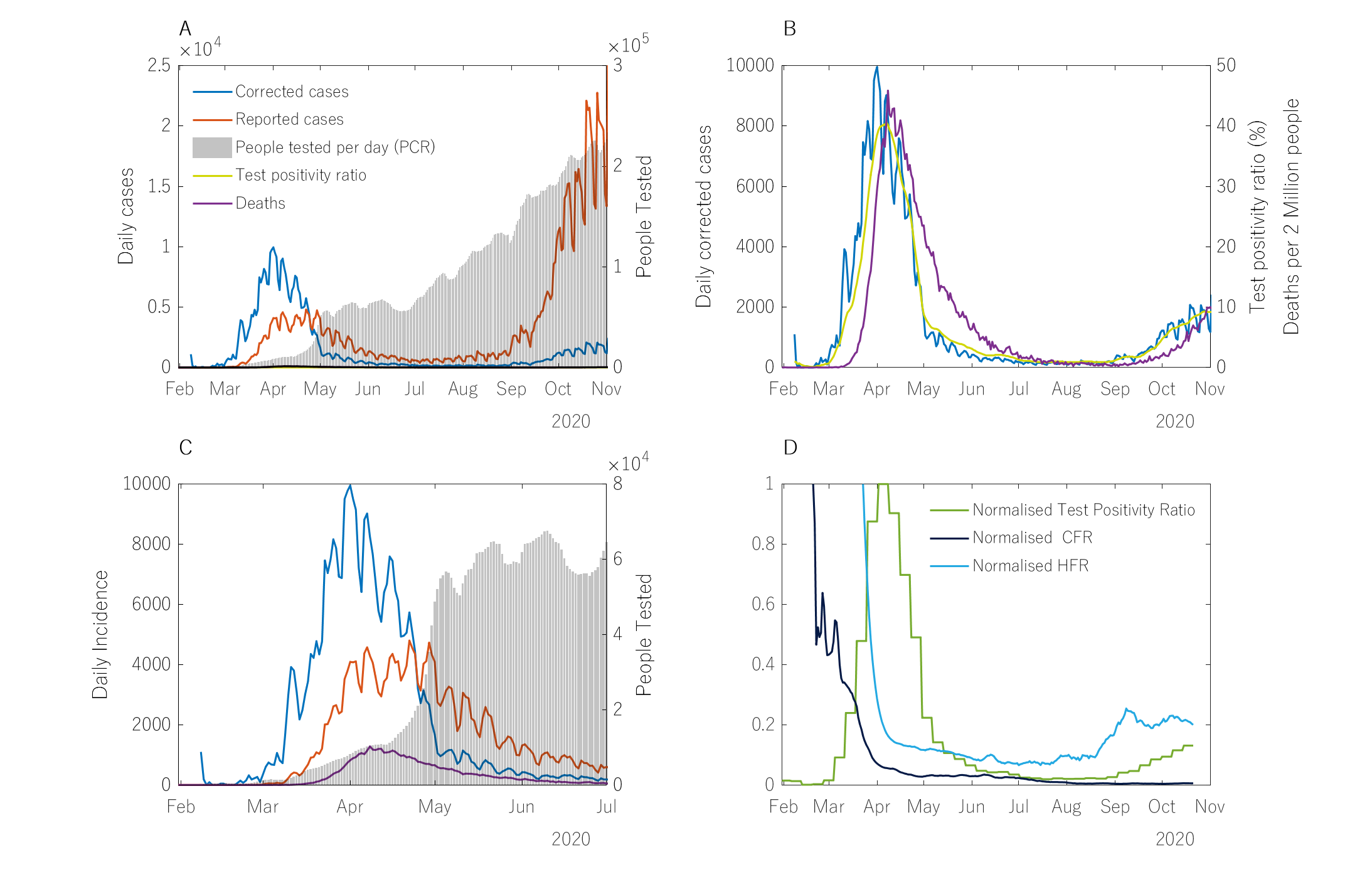

Supplement: S5 Fig — (A) Daily COVID-19 cases and tests in England from Feb 5th 2020 to Nov 7th, 2020, alongside the testing effort corrected case curve. Case correction was done by taking the number of daily tests done on May 1st and extrapolating the number of daily cases that would be reported if the testing effort had been constant over time, i.e., how many daily cases would be reported if 20,000 tests has been done every day. (B) Comparison of testing effort corrected case incidence (blue), test positivity ratio (yellow) and daily deaths per 2 million people (purple). (C) Daily reported incidence of cases, deaths and people tested up to July 1st, 2020. Note the different scale for mortality data used on panels (B) and (C). In panel (C) we present the absolute number of deaths reported per day as a means of comparing its scale to the reported case data. In panel (B) we modify the mortality incidence scale to more easily compared its shape over time against that of the daily corrected cases and test positivity ratio curves. (D) Normalized case fatality ratio (CFR), hospital fatality ratio (HFR) and PCR test positivity ratio (yellow, blue, and green lines, respectively). We assumed fixed time lags of δp = 14 days between PCR testing and death and δh = 12 days between PCR testing and hospitalization. (TIFF) [file pcbi.1009436.s005.tiff]

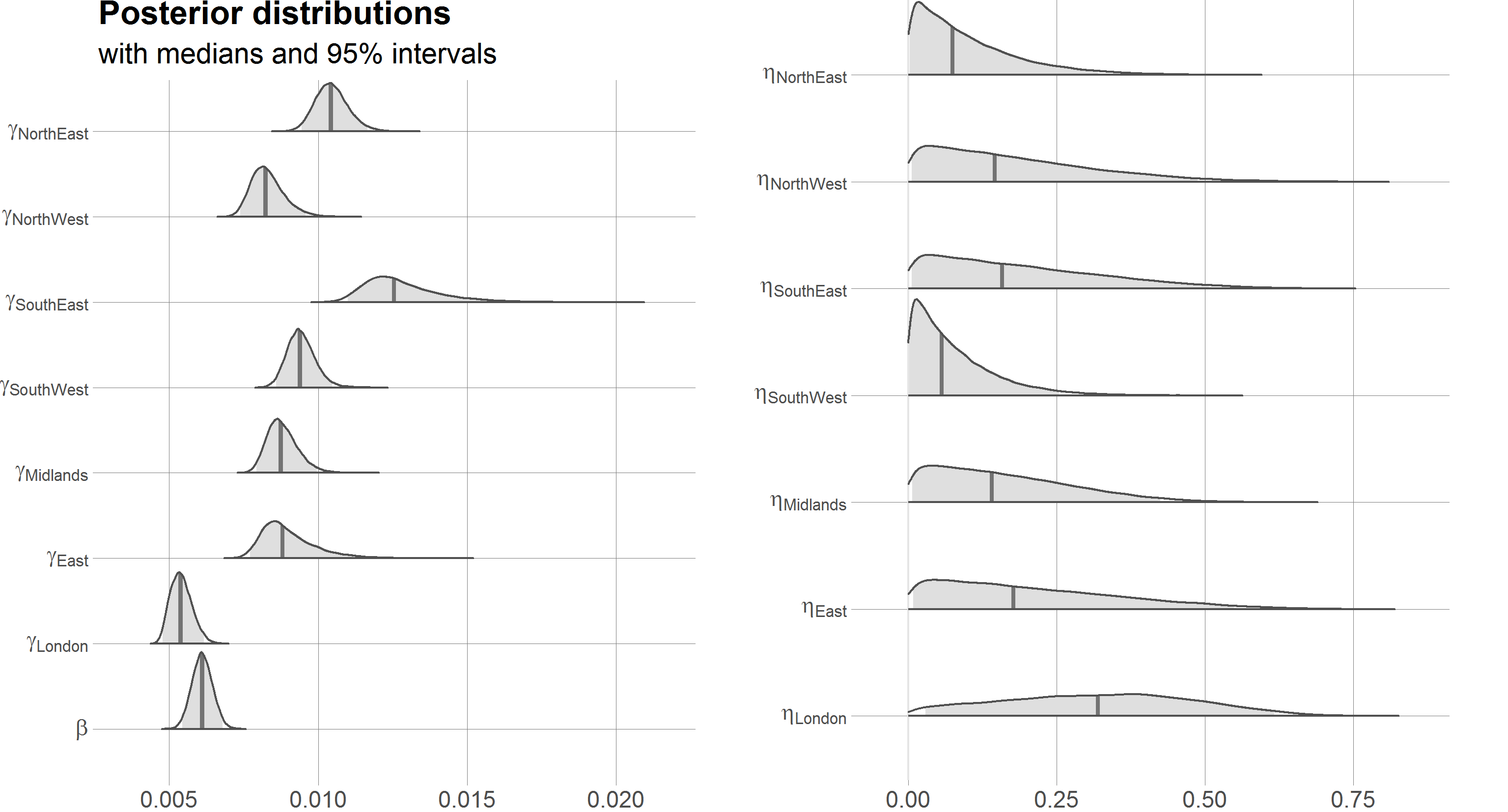

Supplement: S6 Fig — The vertical lines show the median distributions, and the grey shaded regions show the 95% CrI. (TIFF) [file pcbi.1009436.s006.tiff]

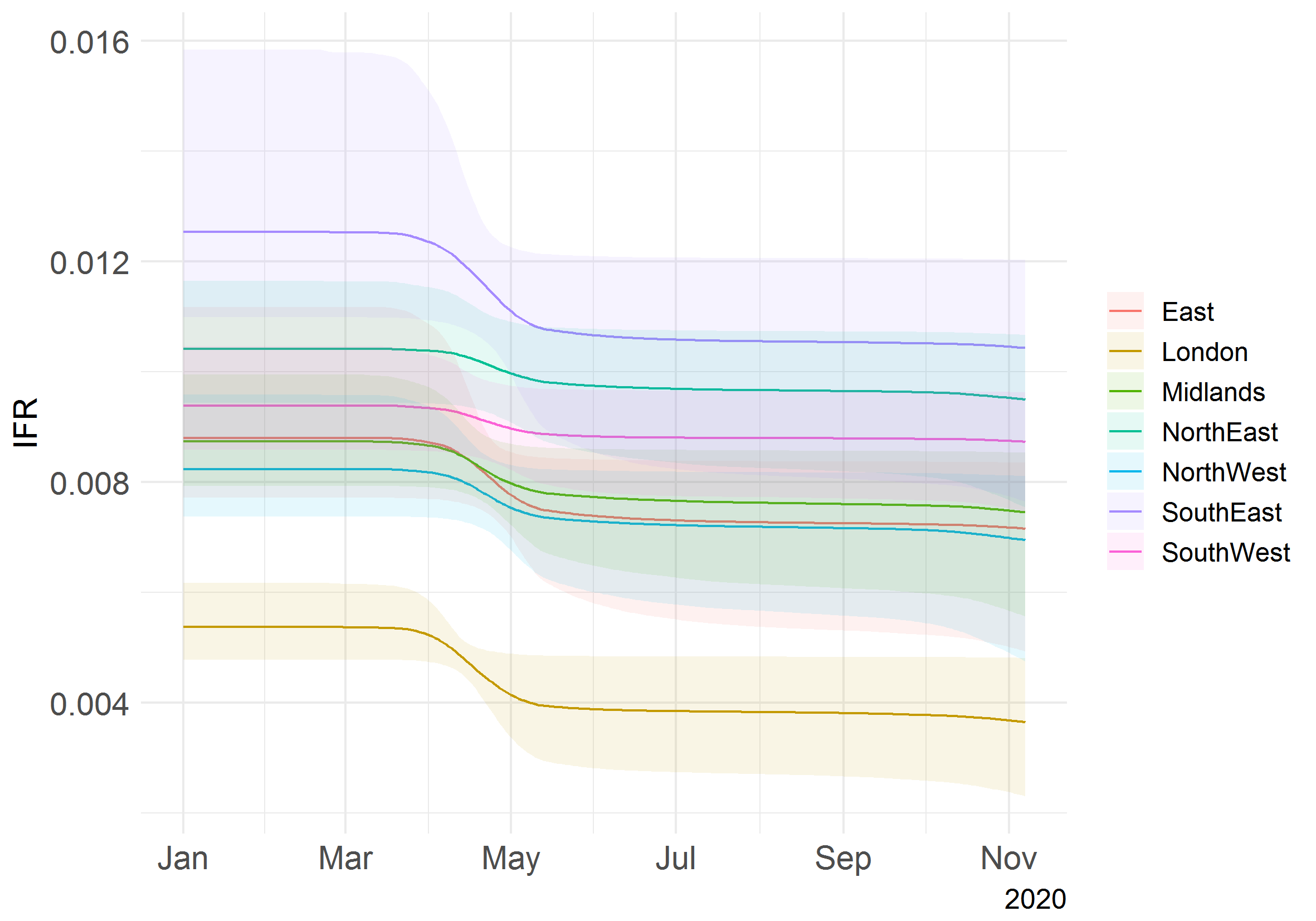

Supplement: S7 Fig — The solid lines show the medians and the shaded regions show the 95% CrI. (TIFF) [file pcbi.1009436.s007.tiff]

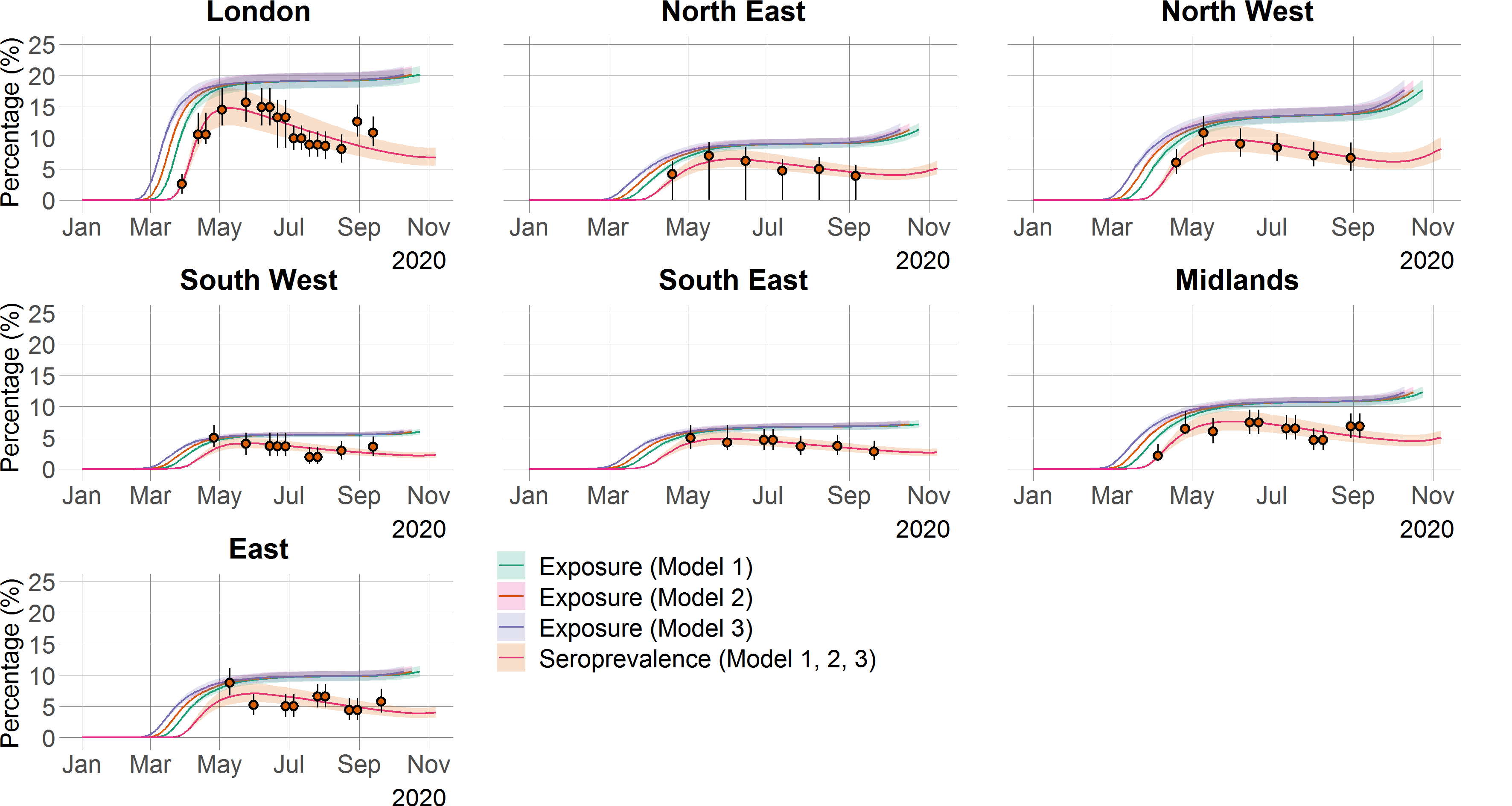

Supplement: S8 Fig — The orange solid circles and black error bars in each regional panel represent the observed seroprevalence data and their credible intervals after adjusting for the sensitivity and specificity of the antibody test. The green, red and purple lines show the median constant IFR model predictions for exposure assuming δϵ as 2, 3 and 4 weeks, respectively, while the shaded regions correspond to the 95% CrI. The green lines show the median constant IFR model predictions for seroprevalence while the shaded regions correspond to the 95% CrI. See S4 Table for details on each model’s assumptions. (TIFF) [file pcbi.1009436.s008.tiff]

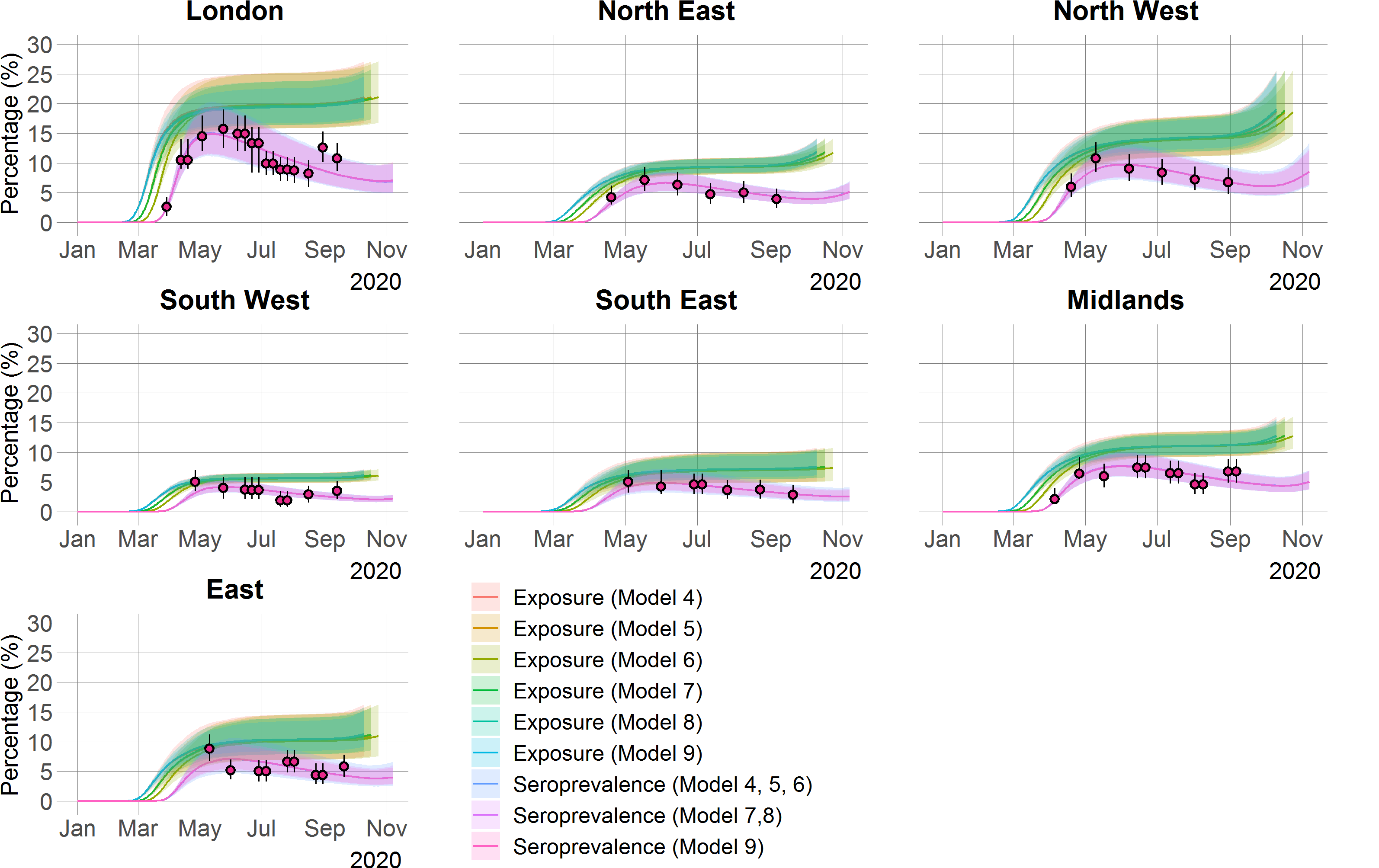

Supplement: S9 Fig — The orange solid circles and black error bars in each regional panel represent the observed seroprevalence data and their credible intervals after adjusting for the sensitivity and specificity of the antibody test. The lines in red, green, and blue tones show the median constant IFR model predictions for exposure assuming δϵ as 2, 3 and 4 weeks, respectively, while the shaded regions correspond to their 95% CrI. The purple lines show the median constant IFR model predictions for seroprevalence while the shaded regions correspond to the 95% CrI. See S4 Table for details on each model’s assumptions. (TIFF) [file pcbi.1009436.s009.tiff]

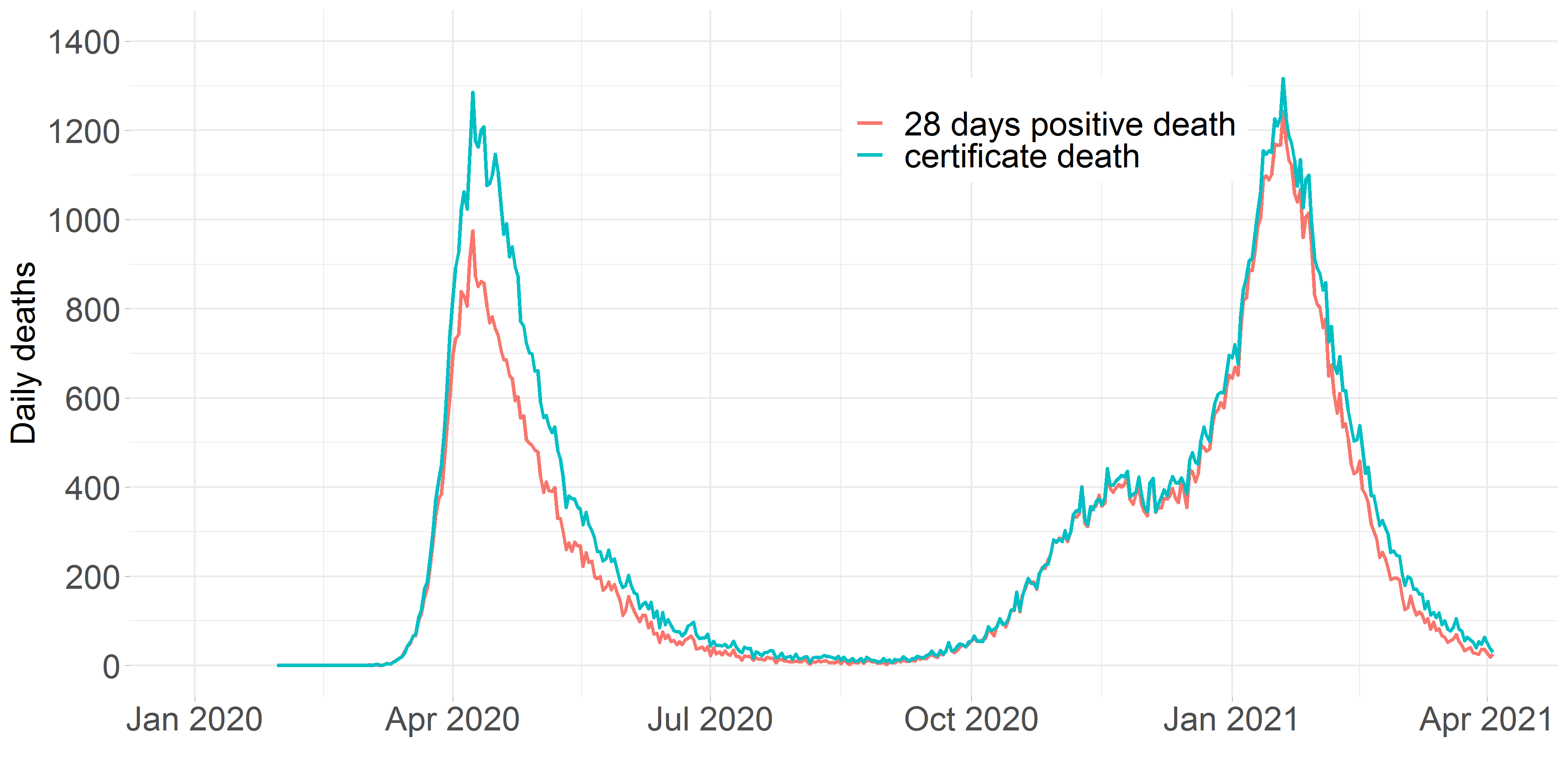

Supplement: S10 Fig — (TIFF) [file pcbi.1009436.s010.tiff]

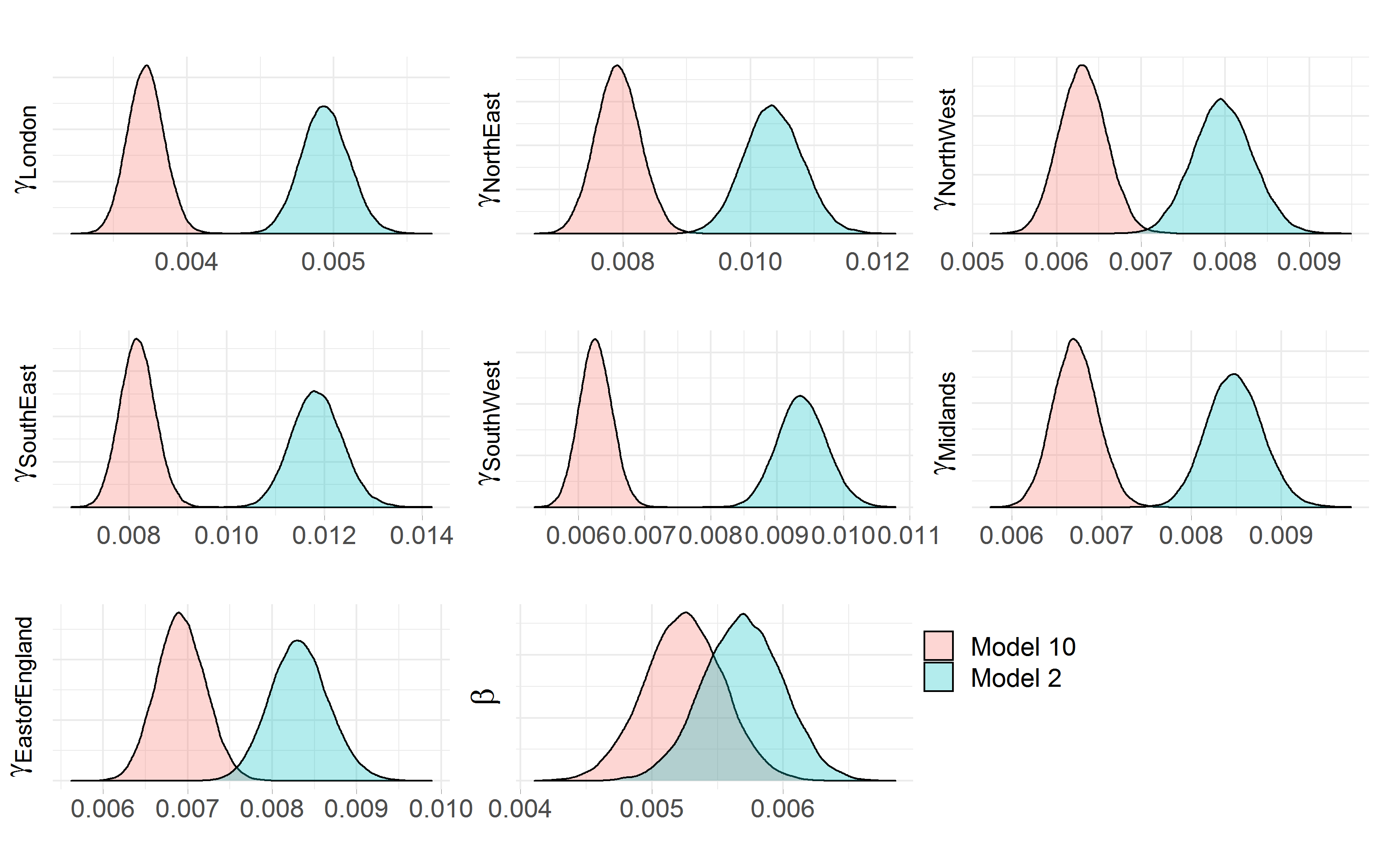

Supplement: S11 Fig — The red regions show the posterior distributions for parameters using deaths within 28 days of positive test as model inputs while the blue regions show the posterior distributions of parameters using death certificate data as model inputs. See S4 Table for details on each model’s assumptions. (TIFF) [file pcbi.1009436.s011.tiff]

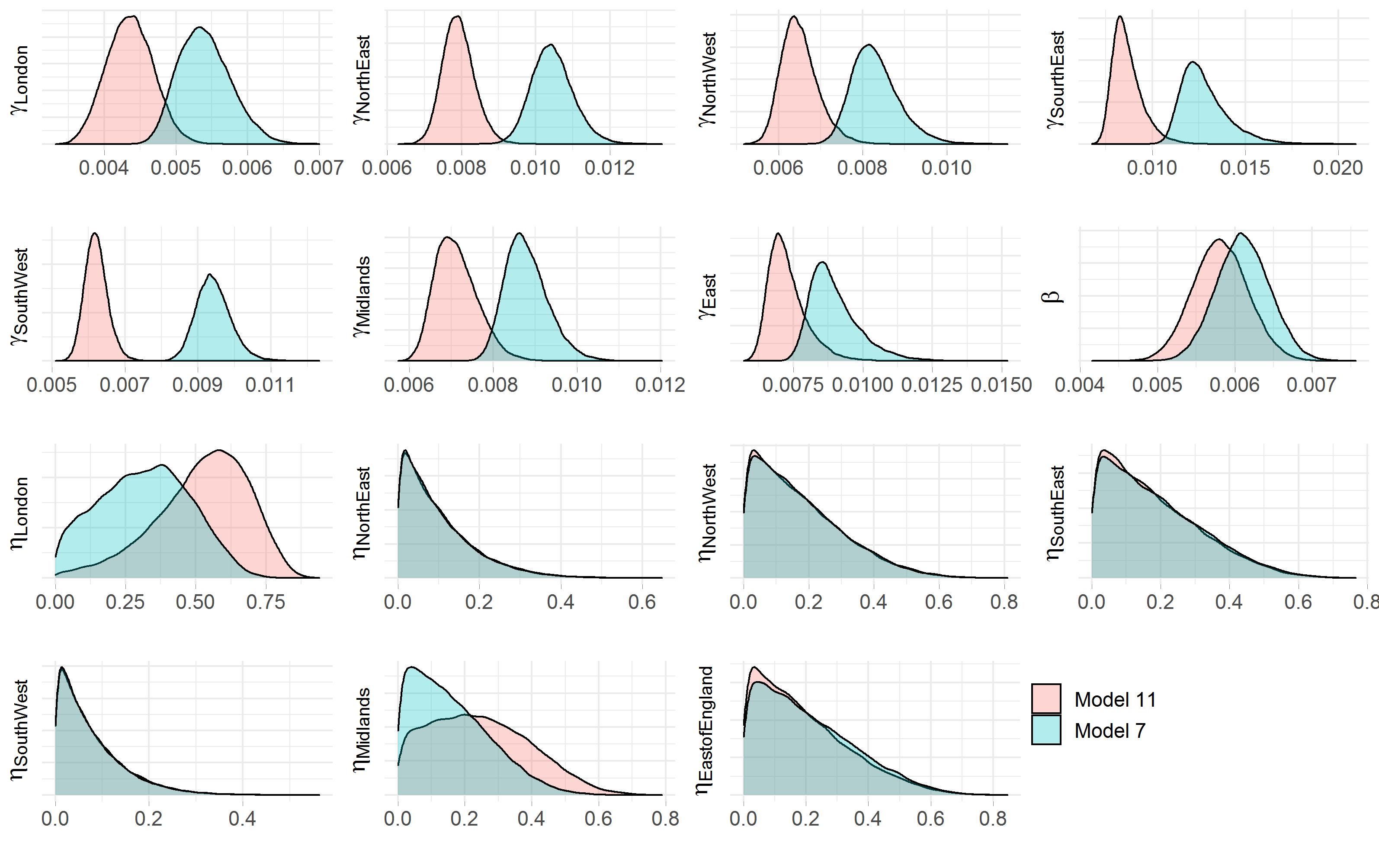

Supplement: S12 Fig — The red regions show the posterior distributions for parameters using deaths within 28 days of positive test as model inputs while the blue regions show the posterior distributions of parameters using death certificate data as model inputs. See S4 Table for details on each model’s assumptions. (TIFF) [file pcbi.1009436.s012.tiff]

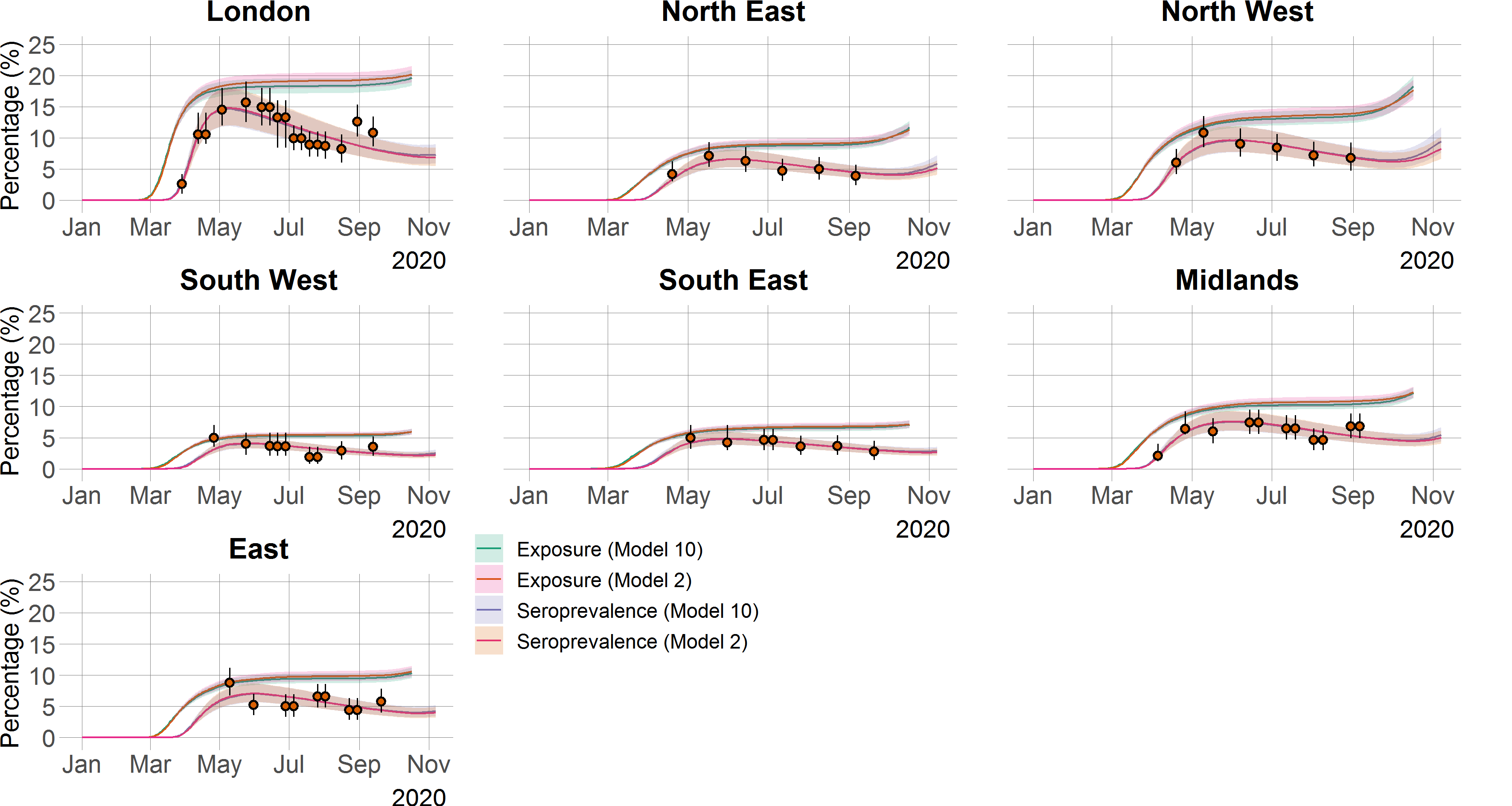

Supplement: S13 Fig — The solid orange circles and black error bars in each regional panel represent the observed seroprevalence data and their credible intervals after adjusting for the sensitivity and specificity of the antibody test. The green and pink lines show the median constant IFR model predictions for exposure using death within 28 days of a positive test and death certificate data as model inputs, respectively, while the shaded regions correspond to the 95% CrIs. The purple and orange lines show the median constant IFR model predictions for seroprevalence using death within 28 days of a positive and death certificate data as model inputs, respectively, while the shaded regions correspond to the 95% CrIs. See S4 Table for details on each model’s assumptions. (TIFF) [file pcbi.1009436.s013.tiff]

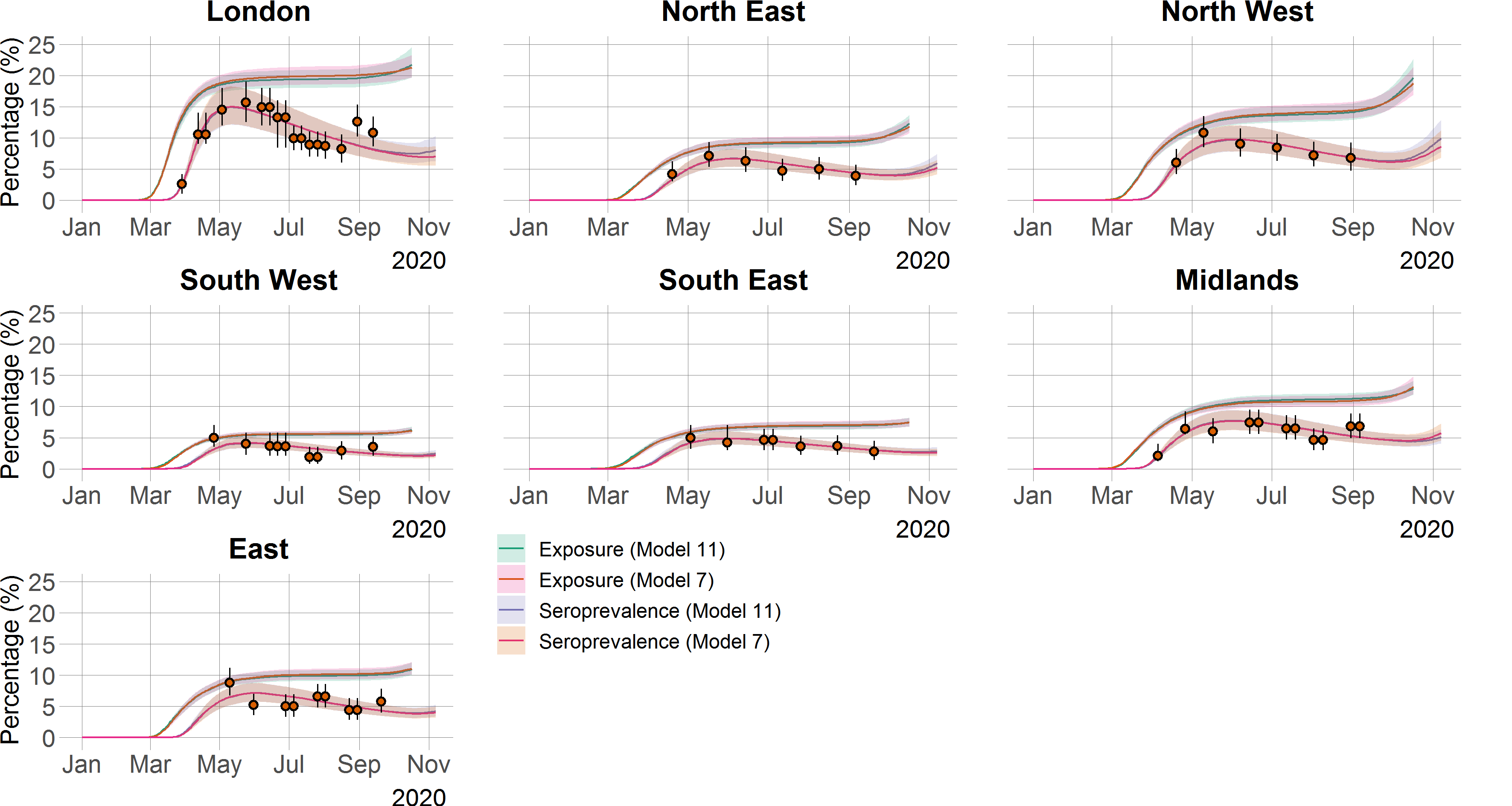

Supplement: S14 Fig — The solid orange circles and black error bars in each regional panel represent the observed seroprevalence data and their credible intervals after adjusting for the sensitivity and specificity of the antibody test. The green and pink lines show the median constant IFR model predictions for exposure using death within 28 days of a positive test and death certificate data as model inputs, respectively, while the shaded regions correspond to the 95% CrIs. The purple and orange lines show the median constant IFR model predictions for seroprevalence using death within 28 days of a positive and death certificate data as model inputs, respectively, while the shaded regions correspond to the 95% CrIs. See S4 Table for details on each model’s assumptions. (TIFF) [file pcbi.1009436.s014.tiff]

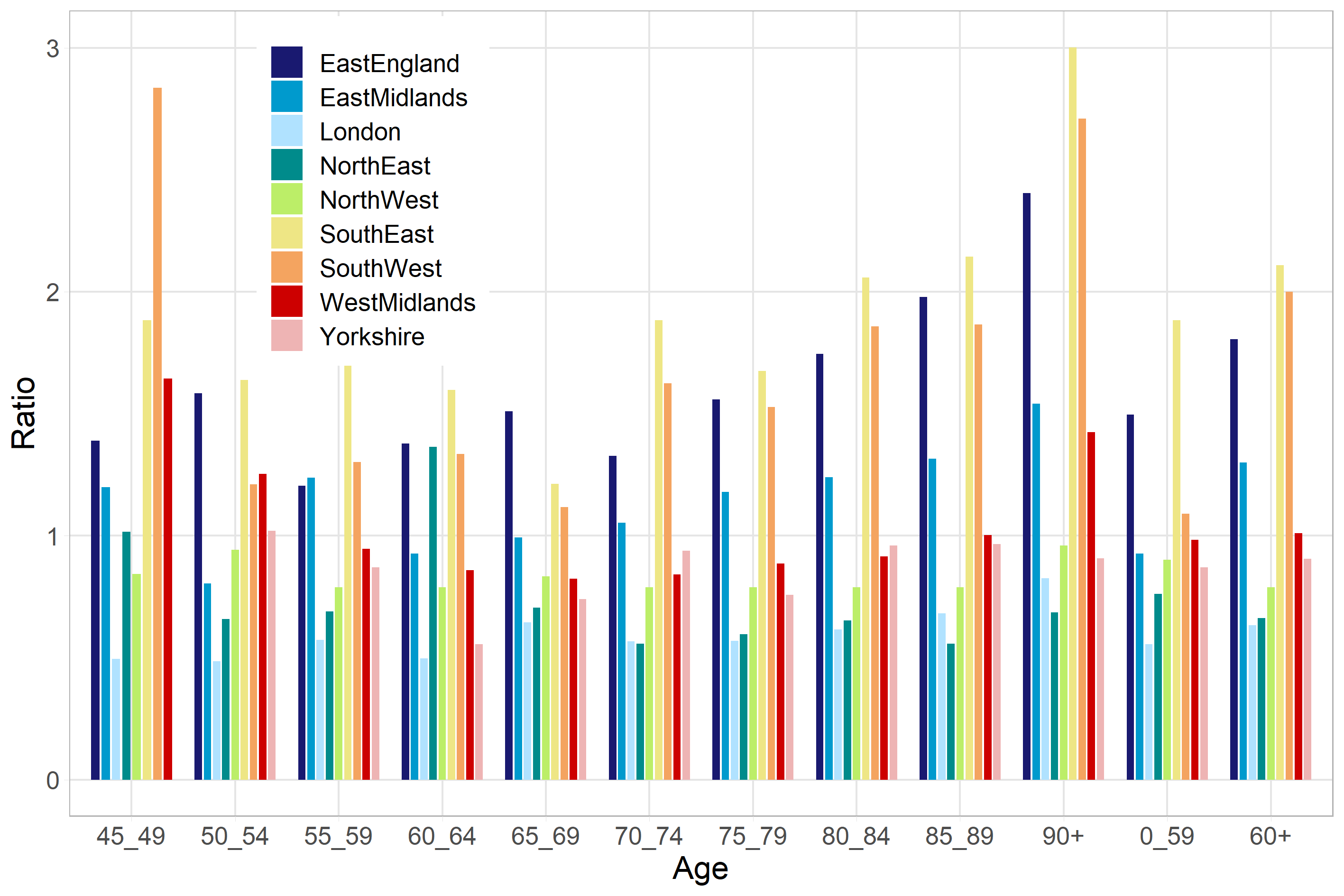

Supplement: S15 Fig — A ratio greater than 1 means that the age specific rate of death was greater in the winter wave than in the preceding spring wave, and vice-versa. (TIFF) [file pcbi.1009436.s015.tiff]

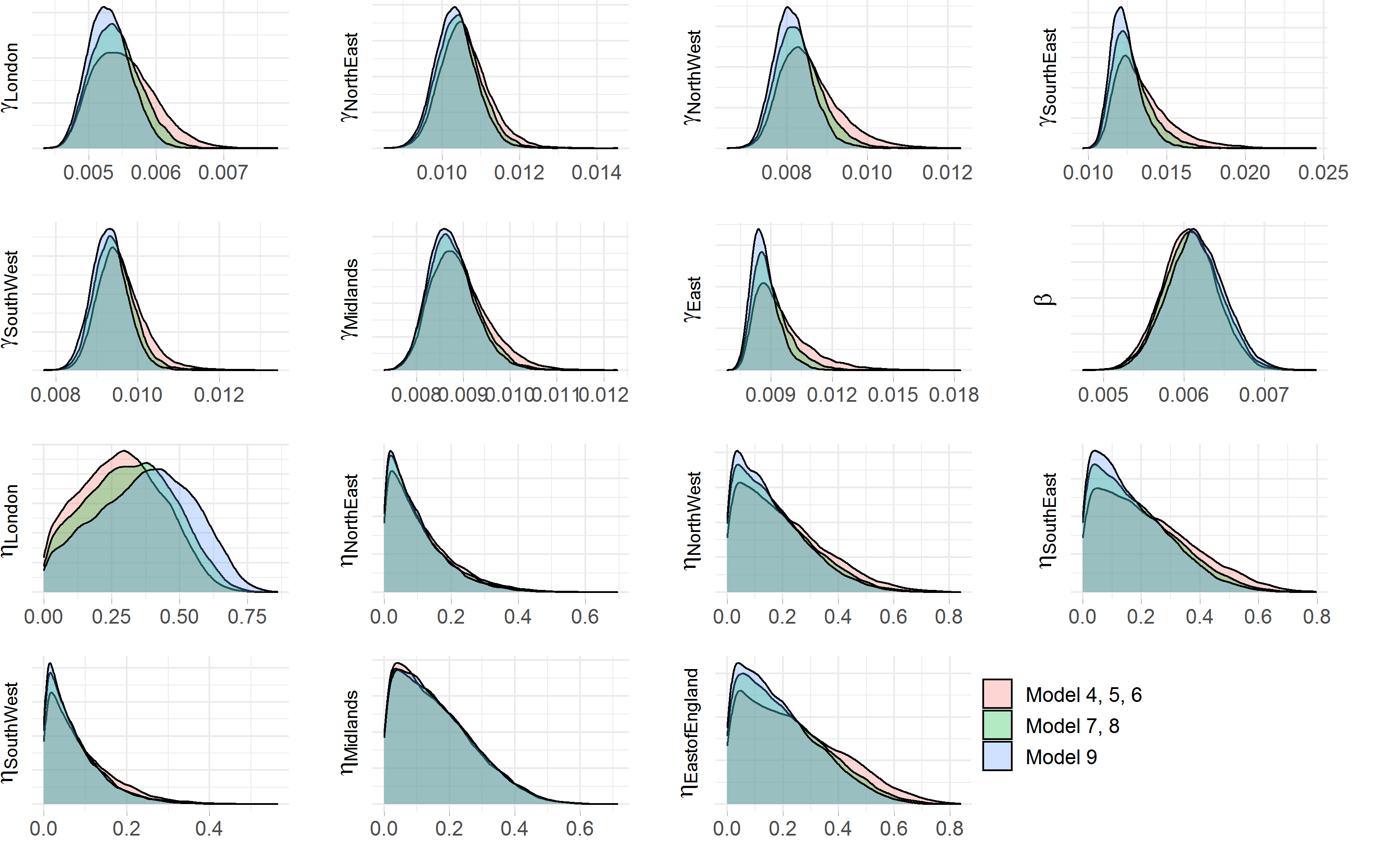

Supplement: S16 Fig — See S4 Table for details on each model’s assumptions. (TIFF) [file pcbi.1009436.s016.tiff]

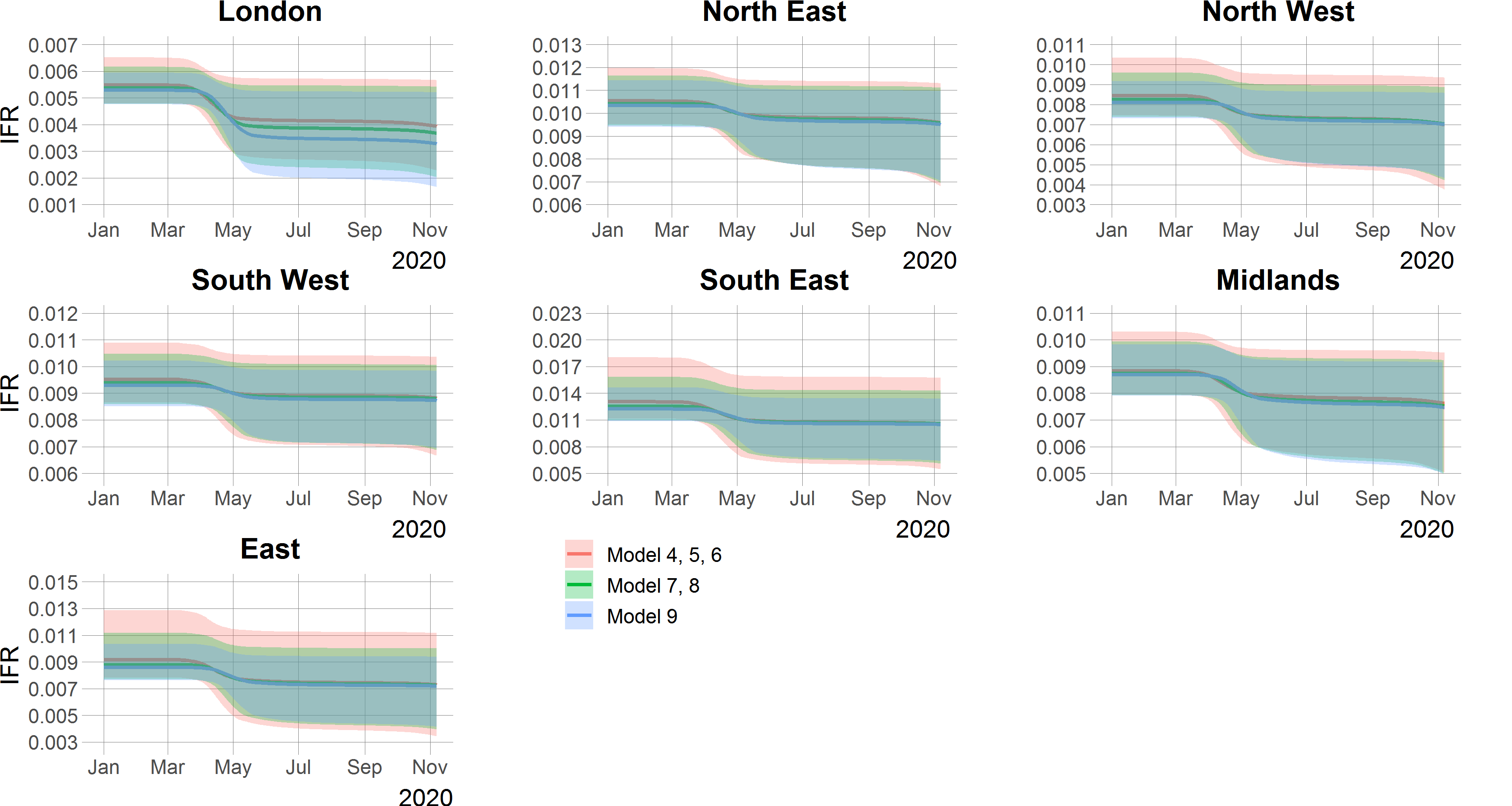

Supplement: S17 Fig — See S4 Table for details on each model’s assumptions. (TIFF) [file pcbi.1009436.s017.tiff]
