# Supplementary material for: Levels of SARS-CoV-2 population exposure are considerably higher than suggested by seroprevalence surveys
Source: PLoS Comput Biol. 2021 Sep 20;17(9):e1009436. doi: 10.1371/journal.pcbi.1009436 (PMC8483393; doi:10.1371/journal.pcbi.1009436)
Supplement: S1 Table — (DOCX) [file pcbi.1009436.s018.docx]

| Parameter | $n_{eff}$ | $\hat{R}$ |
| --- | --- | --- |
| $\beta$ | 12410 | 1 |
| $\gamma_{London}$ | 18054 | 1 |
| $\gamma_{NorthEast}$ | 29625 | 1 |
| $\gamma_{SouthEast}$ | 23952 | 1 |
| $\gamma_{NorthWest}$ | 28611 | 1 |
| $\gamma_{SouthWest}$ | 22006 | 1 |
| $\gamma_{Midlands}$ | 21334 | 1 |
| $\gamma_{East}$ | 20992 | 1 |
