# Supplementary material for: Levels of SARS-CoV-2 population exposure are considerably higher than suggested by seroprevalence surveys
Source: PLoS Comput Biol. 2021 Sep 20;17(9):e1009436. doi: 10.1371/journal.pcbi.1009436 (PMC8483393; doi:10.1371/journal.pcbi.1009436)
Supplement: S2 Table — (DOCX) [file pcbi.1009436.s019.docx]

| Parameter | $n_{eff}$ | $\hat{R}$ |
| --- | --- | --- |
| $\beta$ | 35229 | 1 |
| $\gamma_{London}$ | 32184 | 1 |
| $\eta_{London}$ | 26680 | 1 |
| $\gamma_{NorthEast}$ | 38159 | 1 |
| $\eta_{NorthEast}$ | 33166 | 1 |
| $\gamma_{SouthEast}$ | 24036 | 1 |
| $\eta_{SouthEast}$ | 24430 | 1 |
| $\gamma_{NorthWest}$ | 32887 | 1 |
| $\eta_{NorthWest}$ | 29965 | 1 |
| $\gamma_{SouthWest}$ | 24430 | 1 |
| $\eta_{SouthWest}$ | 31047 | 1 |
| $\gamma_{Midlands}$ | 31943 | 1 |
| $\eta_{Midlands}$ | 27558 | 1 |
| $\gamma_{East}$ | 24703 | 1 |
| $\eta_{East}$ | 25506 | 1 |
