# Supplementary material for: Levels of SARS-CoV-2 population exposure are considerably higher than suggested by seroprevalence surveys
Source: PLoS Comput Biol. 2021 Sep 20;17(9):e1009436. doi: 10.1371/journal.pcbi.1009436 (PMC8483393; doi:10.1371/journal.pcbi.1009436)
Supplement: S3 Table — (DOCX) [file pcbi.1009436.s020.docx]

| Parameter | Median (95% CrI) |
| --- | --- |
| $\beta$ | 0.0061 (0.0054-0.0068) |
| $\gamma_{London}$ | 0.0054 (0.0048-0.0063) |
| $\eta_{London}$ | 0.30 (0.26–0.60) |
| $\gamma_{NorthEast}$ | 0.011 (0.0095–0.012) |
| $\eta_{NorthEast}$ | 0.077 (0.0029–0.33) |
| $\gamma_{NorthWest}$ | 0.0083 (0.0074–0.0098) |
| $\eta_{NorthWest}$ | 0.15 (0.0069–0.51) |
| $\gamma_{SouthWest}$ | 0.0094 (0.0086–0.011) |
| $\eta_{SouthWest}$ | 0.060 (0.0021–0.26) |
| $\gamma_{SouthEast}$ | 0.0013 (0.011-0.017) |
| $\eta_{SouthEast}$ | 0.17 (0.0070–0.53) |
| $\gamma_{Midlands}$ | 0.0088 (0.0079–0.010) |
| $\eta_{Midlands}$ | 0.14 (0.0060–0.42) |
| $\gamma_{East}$ | 0.0089 (0.0077–0.012) |
| $\eta_{East}$ | 0.18 (0.0080–0.57) |
