# Supplementary material for: Levels of SARS-CoV-2 population exposure are considerably higher than suggested by seroprevalence surveys
Source: PLoS Comput Biol. 2021 Sep 20;17(9):e1009436. doi: 10.1371/journal.pcbi.1009436 (PMC8483393; doi:10.1371/journal.pcbi.1009436)
Supplement: S4 Table — Figs 2 and 3 of the main text were generated using Models 2 and 7 respectively. (DOCX) [file pcbi.1009436.s021.docx]

| *Model* | $\delta_{p}$ | $\delta_{\epsilon}$ | *Prior for*$\beta$ | IFR | *death input* |
| --- | --- | --- | --- | --- | --- |
| 1 | N/A | 14 | Uniform | Constant | Death certificate |
| 2 | N/A | 21 | Uniform | Constant | Death certificate |
| 3 | N/A | 28 | Uniform | Constant | Death certificate |
| 4 | 7 | 14 | Uniform | Time-varying | Death certificate |
| 5 | 7 | 21 | Uniform | Time-varying | Death certificate |
| 6 | 7 | 28 | Uniform | Time-varying | Death certificate |
| 7 | 14 | 21 | Uniform | Time-varying | Death certificate |
| 8 | 14 | 28 | Uniform | Time-varying | Death certificate |
| 9 | 21 | 28 | Uniform | Time-varying | Death certificate |
| 10 | N/A | 21 | Uniform | Constant | 28 days positive death |
| 11 | 14 | 21 | Uniform | Time-varying | 28 days positive death |
| 12 | N/A | 21 | Weibull | Constant | Death certificate |
| 13 | 14 | 21 | Weibull | Time-varying | Death certificate |
