# Supplementary material for: Levels of SARS-CoV-2 population exposure are considerably higher than suggested by seroprevalence surveys
Source: PLoS Comput Biol. 2021 Sep 20;17(9):e1009436. doi: 10.1371/journal.pcbi.1009436 (PMC8483393; doi:10.1371/journal.pcbi.1009436)
Supplement: S5 Table — (DOCX) [file pcbi.1009436.s022.docx]

| ***Parameters*** | ***Model***  ***1, 2, 3*** | ***Model***  ***4, 5, 6*** | ***Model***  ***7, 8*** | ***Model***  ***9*** | ***Model***  ***10*** | ***Model***  ***11*** | ***Model***  ***12*** | ***Model***  ***13*** |
| --- | --- | --- | --- | --- | --- | --- | --- | --- |
| $\beta$ | 0.0057  (0.0051,0.0063) | 0.0061  (0.0054 ,0.0067) | 0.0061  (0.0054,0.0068) | 0.0061  (0.0055,0.0069) | 0.0052  (0.0046,0.0058) | 0.0058  (0.0051,0.0065) | 0.0057  (0.0051,0.0063) | 0.0062  (0.0055,0.0068) |
| $\gamma_{London}$ | 0.0049  (0.0046,0.0053) | 0.0055  (0.0048,0.0065) | 0.0054  (0.0048,0.0062) | 0.0053  (0.0048,0.0059) | 0.0037  (0.0035,0.0040) | 0.0044  (0.0037,0.0050) | 0.0049  (0.0046,0.0052) | 0.0054  (0.0048,0.0062) |
| $\gamma_{North West}$ | 0.0080  (0.0073,0.0087) | 0.0084  (0.0075 ,0.010) | 0.0083  (0.0074,0.0098) | 0.0081  (0.0073,0.0092) | 0.0063  (0.0058,0.0069) | 0.0065  (0.0058,0.0075) | 0.0079  (0.0073,0.0086) | 0.0082  (0.0074,0.0096) |
| $\gamma_{North East}$ | 0.010  (0.0095,0.011) | 0.011  (0.0095,0.012) | 0.010  (0.0095,0.012) | 0.010  (0.0094,0.011) | 0.0079  (0.0073,0.0086) | 0.0079  (0.0071,0.0088) | 0.010  (0.0095,0.011) | 0.010  (0.0094,0.012) |
| $\gamma_{South East}$ | 0.012  (0.011,0.013) | 0.013  (0.011,0.018) | 0.013  (0.011,0.017) | 0.012  (0.011,0.015) | 0.0082  (0.0075,0.0089) | 0.0085  (0.0075,0.011) | 0.012  (0.011,0.013) | 0.013  (0.011,0.016) |
| $\gamma_{South West}$ | 0.0094  (0.0087,0.010) | 0.0095  (0.0087,0.011) | 0.0094  (0.0086,0.011) | 0.0093  (0.0085,0.010) | 0.0063  (0.0058,0.0067) | 0.0062  (0.0057,0.0069) | 0.0093  (0.0086,0.010) | 0.0094  (0.0086,0.010) |
| $\gamma_{Midlands}$ | 0.0085  (0.0079,0.0091) | 0.0088  (0.0079,0.010) | 0.0088  (0.0079,0.010) | 0.0087  (0.0079,0.0098) | 0.0067  (0.0062,0.0072) | 0.0071  (0.0063,0.0082) | 0.0085  (0.0079,0.0091) | 0.0087  (0.0079,0.010) |
| $\gamma_{East}$ | 0.0083  (0.0077,0.0090) | 0.0092  (0.0078,0.013) | 0.0089  (0.0078,0.012) | 0.0086  (0.0076,0.010) | 0.0069  (0.0064,0.0075) | 0.0072  (0.0063,0.0090) | 0.0083  (0.0076,0.0090) | 0.0087  (0.0077,0.011) |
| $\eta_{London}$ | N/A | 0.28  (0.021,0.57) | 0.32  (0.028,0.62) | 0.38  (0.033,0.68) | N/A | 0.54  (0.14,0.79) | N/A | 0.33  (0.033,0.62) |
| $\eta_{North West}$ | N/A | 0.17  (0.0086,0.56) | 0.15  (0.0059,0.49) | 0.13  (0.0058,0.46) | N/A | 0.14  (0.0057,0.49) | N/A | 0.15  (0.0061,0.49) |
| $\eta_{North East}$ | N/A | 0.084  (0.0029,0.35) | 0.074  (0.0031,0.31) | 0.073  (0.0030,0.31) | N/A | 0.076  (0.0028,0.32) | N/A | 0.075  (0.0031,0.32) |
| $\eta_{South East}$ | N/A | 0.19  (0.0084,0.58) | 0.17  (0.0069,0.53) | 0.14  (0.0064,0.47) | N/A | 0.15  (0.0071,0.50) | N/A | 0.16  (0.0068,0.51) |
| $\eta_{South West}$ | N/A | 0.066  (0.0025,0.29) | 0.058  (0.0020,0.25) | 0.053  (0.0020,0.24) | N/A | 0.058  (0.0022,0.25) | N/A | 0.057  (0.0022,0.26) |
| $\eta_{Midlands}$ | N/A | 0.14  (0.0067,0.42) | 0.40  (0.0064,0.13) | 0.14  (0.0065,0.43) | N/A | 0.23  (0.014,0.55) | N/A | 0.14  (0.0070,0.43) |
| $\eta_{East}$ | N/A | 0.21  (0.0096,0.62) | 0.19  (0.0085,0.57) | 0.16  (0.0071,0.51) | N/A | 0.16  (0.0062,0.54) | N/A | 0.18  (0.0078,0.55) |
