# Supplementary material for: Levels of SARS-CoV-2 population exposure are considerably higher than suggested by seroprevalence surveys
Source: PLoS Comput Biol. 2021 Sep 20;17(9):e1009436. doi: 10.1371/journal.pcbi.1009436 (PMC8483393; doi:10.1371/journal.pcbi.1009436)
Supplement: S6 Table. Linear regression models exploring relationships between demographic and epidemiological factors and estimated regional IFRs. Each row refers to a unique linear regression model and indicates which covariate was used, alongside the resulting slope and intercept estimates (with accompanying — (DOCX) [file pcbi.1009436.s023.docx]

| Independent Variable | Slope | Intercept | *p-value* |
| --- | --- | --- | --- |
| Proportion of people over 45 years of age | **0.0382**  [0.00292, 0.0735] | **-0.00819**  [-0.0239, 0.00751] | *0.0388* |
| Proportion of people over 60 years of age | **0.0440**  [0.000193, 0.0879] | **0.001936**  [0.0127, 0.00880] | *0.0493* |
| Deaths in the community relative to deaths in care homes | **-0.000787**  [-0.00128, -0.000290] | **0.0125**  [0.00987, 0.0151] | *0.00962* |
| Care home beds per 100 people over 75 years of age | **0.00147**  [0.000220, 0.00272] | **-0.00450**  [-0.0168, 0.00677] | *0.0293* |
| Diabetes prevalence | **-0.000587**  [-0.00574, 0.00457] | **0.0128880**  [-0.0236, 0.0494] | *0.781* |
| Chronic liver disease mortality (per 100,000) | **8.81e-05**  [-0.000650, 0.000826] | **0.00764**  [-0.00187, 0.0172] | *0.771* |
| Chronic obstructive pulmonary disease mortality (per 100,000) | **1.28e-05**  [-0.000235, 0.000261] | **0.00810e-03**  [-0.00461, 0.0208] | *0.899* |
