# Supplementary material for: Levels of SARS-CoV-2 population exposure are considerably higher than suggested by seroprevalence surveys
Source: PLoS Comput Biol. 2021 Sep 20;17(9):e1009436. doi: 10.1371/journal.pcbi.1009436 (PMC8483393; doi:10.1371/journal.pcbi.1009436)
Supplement: Attachment — Submitted filename: Response_to_reviewers.docx [file pcbi.1009436.s024.docx]

**Reply to reviewers’ comments**, **submission PCOMPBIOL-D-21-00584**:

"Levels of SARS-CoV-2 population exposure are considerably higher than suggested by seroprevalence surveys"

**Editor’s summary:**

i) ***the need to discuss the sensitivity of the proposed approach to key assumptions; simple simulations under different scenarios could add to the assessment of the robustness of the proposed correction methodology.***

**Author response:** We have performed sensitivity analyses to evaluate the robustness of the proposed methodology. Please see our responses to **comments 1.5 - 1.7** and **1.9**.

ii) ***the need to adjust the topics in the discussion section to the relevant material actually dealt with in the manuscript.***

**Author response:** We appreciate there was some dissonance amongst the reviewers on how the main message was conveyed and transpired from the narrative in the initial submission. We have made some changes to the result presentation, quantification of potential infection fatality ratio (IFR) drivers and discussion outline to, hopefully, provide more clarity on the main conclusions in the paper. Please see our responses to **comments 3.7** and **4.1**.

***iii) the need to add references supporting the claims invoked along with the modeling exercise.***

**Author response:** We have made a concerted effort to provide references for all data and literature results mentioned throughout, at the risk of being repetitive, according to **comments 3.10** and **3.13**.

**Author’s note:**

We appreciate the reviewers’ constructive comments and feel the revised manuscript should clarify all the concerns raised and be a lot clearer to a wider audience. All new sources of data and code used to generate the results in the detailed response to reviewer’s comments below can be found in the github repository that accompanied the original submission: <https://github.com/SiyuChenOxf/COVID19SeroModel/tree/master>.

**Reviewer 1**

1.1. ***This is a really nicely written, concise and well conducted study. It does have a number of methodological similarities to the cited Imperial College report on a similar topic, but in my opinion, this is a nice pared-down version that provides some new and interesting insights. I feel the analyses and most of the text are already very polished, so none of my comments are particularly critical. I would just suggest a few clarifications, additional discussion points and optional sensitivity analyses around the fixed parameter assumptions.***

***The shared code looks good and is well documented, thanks!***

**Author response:** We appreciate the reviewer’s positive feedback and are very happy to respond to their comments.

1.2. ***The discussion section is very brief and should include limitations and discuss sensitivity to key assumptions (namely the reliance on death data as absolute truth, though see below). For example, for the English data, the IFR will likely change depending on whether “deaths within 28 days of a positive test” or “deaths with COVID-19 on the death certificate” are used. More specific comments are in the “Minor comments” below, but in general the authors should discuss the study limitations.***

**Author response:** We thank the reviewer for these observations. We’ve expanded the results and discussion sections to address how sensitive the results presented here are to key assumptions such as the source of death data. This is quite a complex issue which has been mostly skewed to investigating under-reporting of deaths in low- and middle-income countries (LMICs). Whilst this is obviously extremely significant, for several reasons, we must not assume that mortality is perfectly reported in high-income countries (HICs). In fact, all HICs relied on a clinical cause of death determination algorithm before their testing capacity reached sufficiently high numbers for all COVID-19 suspected deaths to be investigated by PCR testing. In the UK this translates to 17 thousand more COVID-19 deaths as per the death certificate, when comparing to deaths with a positive PCR test in the 28 days prior, in the 2020 spring wave alone. An argument could be made that the UK has consistently over-reported deaths, since all patients meeting the clinical definition would be labelled COVID-19 patients in the absence of a PCR test in the early stages of the pandemic, and more recently, people dying of other causes whom have had a positive PCR test will also be labelled a COVID-19 death (due to the large numbers of tests per day). To investigate how the definition of death affects the results, we have replicated the model results with the “deaths within 28 days of a positive test” datasets – Figures S6:9 in the new submission. We have also performed a comprehensive sensitivity analysis on our assumptions of a fixed delay between exposure and seroconversion and between PCR test and seroconversion – please see reply to **comment 1.6** below.

1.3. ***The other discussion-based comment I have is on how well age structure can explain the disparate IFR estimates. The authors place a lot of weight on a younger average age explaining the lower IFR in London. This makes sense, but the paper offers no formal analyses to explore the point. Are the other region-level differences in IFR also consistent with differences in age structure? For example, the estimate for the South East is quite a lot higher than the East. Another way to think about it is that previous studies on IFR are often stratified by age group – would combining region-specific age distributions with a single age stratified IFR curve give mean IFRs that are consistent with these estimates, or would there still be unexplained residual differences? I would encourage the authors to think about how they might be able to support their hypothesis quantitatively, either through additional analyses or a more thorough consideration of demographic data. At the very least they should soften the language around “age structure explains these differences” without faithfully discussing caveats and alternative hypotheses.***

**Author response:** We agree with the reviewer that our argument offering age structure as a possible explanation for differences in IFR across regions should have been better substantiated. This was never meant to take away from the main message that serological studies significantly under-estimate the amount of prior exposure in the population. However, two interesting results emerged from the main analyses: (1) baseline IFR estimates across regions in England vary significantly; (2) Only in London does IFR seem to time dependent, decreasing as the pandemic progresses. We posited that differences in population age structures^[1]^ can explain both phenomena and laid out our rationale. The revised document now includes several quantitative analyses of the relationship between age (and age-related variables, such as number of people in care homes) regional structure and estimated IFRs. From **Figure 1** below we can easily partition each region into clusters according to the percentage of people over a pivoting point at age 45. A straightforward linear regression results in 61% of the variance in estimated IFR being explained by the proportion of people in the different regions over the age of 45.

**Figure 1.** Population age distributions by region.

 **Figure 2.** Ratio between relative rates of deaths of people who died within 28 days of their first positive test (per 100,000 population) in the winter wave of 2020 vs. the 2020 spring wave. A ratio greater than 1 means that the age specific rate of death was greater in the winter wave than in the preceding spring wave, and vice-versa.

Further exploration of other explanatory covariates is presented as supplementary information in the revised manuscript. We have put some effort into providing more contextual information and frame the age structure as the most parsimonious hypothesis, but not the definitive explanation of the observed trends. We follow this initial exploration by providing supporting data on age-dependent mortality rates at different stages of the epidemic. This data illustrates how in London mortality rates decreased substantially since the first spring wave, much more so than in any other region – **Figure 2** above. This effect is clear across all ages.

1.4. ***First, the assumption that reported tests represent exactly 100% of true COVID-19-associated deaths. I think this is a fine assumption, but there may be some violations eg. very early in the epidemic not all deaths were tested. To make this framework generalizable to other settings where death reporting and testing is not quite so rigorous as England/the UK, would it be possible to add an additional scaling parameter (possibly time-varying) to the model representing death reporting? This could then be fixed or estimated with an informative prior (eg. the posterior for IFR and beta might include 95% prior probability that death data represents 90%-110% of true deaths. To clarify, this isn’t a change that is necessary for publication, but it may help apply this model to other settings.***

**Author response:** We thank the reviewer for these considerations. Related to the issue raised in the next point, we chose to use the death clinical notification dataset, specifically because of lower testing numbers during the epidemic onset. In reality, one could consider that might even be an over-reporting in countries like the UK, where patients with COVID-19 like symptoms were labelled as SARS-CoV-2 deaths, without any testing to confirm it. Similarly, the current definition for official reported deaths can overestimate the number of deaths actually caused by the virus, by labelling all deaths in the 28 days following a positive test as COVID-19 deaths. We prefer to stay away from such arguments but certainly appreciate the nuances of different countries’ reporting systems. We are fortunate to work with over 40 LMIC countries under the umbrella of the CoMo consortium (https://como.bmj.com/) and have very detailed knowledge of the challenges in inferring ascertainment rates for both cases and deaths. Whilst we could add an additional scaling parameter (possibly time-varying) to represent the reporting rate of deaths, we would end up with an identifiability issue around the true value of IFR, and would only be able to report a compound metric of the relationship between reported mortality, some uncertainty around what the reporting rate of that is, and the uncertainty around the true relationship between the expected number of deaths corrected for under-reporting and the number of infections. We are currently doing research in this space but feel that it is outside the remit of this publication under consideration.

1.5. ***Another consideration (which would be interesting in the publication) might be to re-fit the model using the “Deaths within 28 days of a positive test” data from the same source to get an idea of how much this affects the estimates. I don’t think you need to get into the politically charged territory of “what is a COVID-19 death?”, but it might have implications for the seroreversion rate.***

**Author response:** This is an interesting point that we had in fact grappled with prior to submission but ultimately considered that our choice of mortality data was the most appropriate. We opted against the “28 days positive death” data since it appears to significantly underestimate COVID-19-associated deaths in the beginning of the pandemic, at a time when the transmission was very high and PCR testing capacity was lowest (Figure 3). Note that once testing capacity reached the tens of thousands of tests per day, the two mortality data streams report essentially the same figures. However, given the interest shown in the sensitivity of the results to said choice, we now include results from fits to the “Deaths within 28 days of a positive test” data as supplementary materials. Overall, the estimated seroreversion rate is ~10% lower (*β* parameter in Figures 4 and 6) and the IFRs are ~15-30% lower (*γ* parameters in Figures 4 and 6) than the original estimates when using “Deaths within 28 days of a positive test” data. This is an expected consequence of having the model fit to the same serology data, whilst assuming there are 17,000 less deaths in the spring 2020 wave. More importantly, the cumulative exposure predictions are extremely robust to the mortality inputs (Figures 5 and 7).

**Figure 3.** Certificate death and 28 days positive death comparison in England. The blue curve shows results for the certificate death while the orange curve shows the 28 days positive death.

**Figure 4.** **Comparison of posterior estimation of parameters in the constant IFR model.** The red regions show the posterior distribution estimation of parameters using 28 days positive deaths as model inputs while the blue area shows the posterior distribution estimation of parameters using certificate death as model inputs.

**Figure 5. Comparison of time course of the SARS-Cov-2 pandemic up to 7 November 2020 for seven regions in England for constant IFR model between using 28 days positive death and certificate death as model inputs.** The orange solid circles and black error bars in each regional panel represent the observed seroprevalence data and its confidence interval after adjusting for the sensitivity and specificity of the antibody test. The green and orange lines show the median constant IFR model predictions for exposure using 28 days positive death and certificate death as model inputs respectively while the shaded areas correspond to 95% CrI. The purple and pink lines show the median constant IFR model predictions for seroprevalence using 28 days positive death and certificate death as model inputs respectively while the shaded areas correspond to 95% CrI.

**Figure 6.** **Comparison of posterior estimation of parameters in the time-varying IFR model.** The red region shows the posterior distribution estimation of parameters using 28 days positive deaths as model inputs while the blue area shows the posterior distribution estimation of parameters using certificate death as model inputs.

**Figure 7. Comparison of time course of the SARS-Cov-2 pandemic up to 7 November 2020 for seven regions in England for time varying IFR model between using 28 days positive death and certificate death as model inputs.** The orange solid circles and black error bars in each regional panel represent the observed seroprevalence data and its confidence interval after adjusting for the sensitivity and specificity of the antibody test. The green and orange lines show the median time varying IFR model predictions for exposure using 28 days positive death and certificate death as model inputs respectively while the shaded areas correspond to 95% CrI. The purple and pink lines show the median time varying IFR model predictions for seroprevalence using 28 days positive death and certificate death as model inputs respectively while the shaded areas correspond to 95% CrI.

1.6. ***Second, the assumption that reporting delays are fixed and constant over time. There are a few parts to this point. One is that the cited studies on the symptoms-to-death and symptoms-to-discharge delays are from China in the early stages of the pandemic. There might be reason to suspect that these delays differ both by region (based on how patients are treated etc) and over time (based on burden on healthcare services). In fact, the assumption of a 14 day value for delta_p does not fully square with the citations. Furthermore, a 2-day delay between onset and PCR test is quite short and is likely an underestimate for periods of high testing demand. These are quite pedantic points because I very much doubt changing the delays by a few days will affect the results, but these are strong assumptions which are likely to be violated in reality. I would suggest discussing the limitations of these. Furthermore, the choice of a fixed delay is a simplification of the delay-distribution approaches used elsewhere, which could also be acknowledged (ie. integrating a gamma distribution of delays rather than using a single value). A way to approach these concerns could be re-running the model under a few different assumptions for the delays to get an idea of whether or not the estimates are affected.***

**Author response:** We appreciate the reviewer’s suggestion and provide a sensitivity analysis on the assumption of delays between exposure and seroconversion and between a PCR test and seroconversion in the revised manuscript as supplementary material. We opted for a discrete parameter space exploration, estimating the time varying IFR model parameters for the combinations of $\delta_{p}$ and $\delta_{\epsilon}$ listed in **Table 1** below. Note that the condition $\delta_{p}<\delta_{\epsilon}$ is a strict requirement. Parameter $\delta_{p}$ does not appear in Equation (1) in the methods section, thus, estimates using the constant IFR model are only sensitive to changes in $\delta_{\epsilon}$ (**Figure 8**). Interestingly, the time varying IFR model is relatively insensitive to $\delta_{p}$ (**Figure 9**) since changes in $\delta_{p}$ have a limited impact on the shape of the Epidemic Stage curve, and consequently on IFR changes over time (**Figure 10**). However, changes in $\delta_{\epsilon}$ will produce a marked shift in the timing of cumulative exposure increase (**Figure 9**). A detailed description of the sensitivity analyses performed is provided in supplementary methods and the relevant results are now shown as supplementary figures. We also clarified that the choice of a fixed delay is a simplification of the delay-distribution approaches used elsewhere.

**Table 1. Combinations of** $\boldsymbol{\delta}_{\boldsymbol{p}}$**and** $\boldsymbol{\delta}_{\boldsymbol{\epsilon}}$ **values explored in the sensitivity analysis of the time varying IFR model.**

| Parameter set | $\delta_{p}$ | $\delta_{\epsilon}$ |
| --- | --- | --- |
| 1 | 14 | 21 |
| 2 | 7 | 28 |
| 3 | 14 | 28 |
| 4 | 21 | 28 |
| 5 | 7 | 21 |
| 6 | 7 | 14 |

**Figure 8. Comparison of time course of the SARS-Cov-2 pandemic up to 7 November 2020 for seven regions in England for constant IFR model given** $\boldsymbol{\delta}_{\boldsymbol{e}}$ **as 14 days, 21 days and 28 days.** The orange solid circles and black error bars in each regional panel represent the observed seroprevalence data and its confidence interval after adjusting for the sensitivity and specificity of the antibody test. The green, orange and purple lines show the median constant IFR model predictions for exposure using $\delta_{\epsilon}$ as 14 days, 21 days and 28 days respectively while the shaded areas correspond to 95% CrI. The pink lines show the median constant IFR model predictions for seroprevalence while the shaded areas correspond to 95% CrI.

**Figure 9. Comparison of time course of the SARS-Cov-2 pandemic up to 7 November 2020 for seven regions in England for time-varying IFR model under the simulation settings in Table 1**.

**Figure 10. Comparison of estimation for IFR for seven regions in England for time-varying IFR model using** $\boldsymbol{\delta}_{\boldsymbol{p}}$ **as 7 days, 14 days and 21 days.** The red, green and blue lines show the IFR estimations using $\delta_{p}$ as 7 days, 14 days and 21 days respectively.

1.7. ***Third, averaging test positivity rates between Pillar 1 and Pillar 2. This feels like quite a crude approach, given how much test positivity can reflect changing coverage in the different test populations as well as the state of the epidemic. Again, I suspect this won’t affect the results too much, but I do wonder if things might change if Pillar 1 OR 2 were used. Another option might be to use PCR prevalence data from REACT or ONS, which albeit not incidence either, will be unbiased by changing testing capacity and coverage.***

**Author response:** We thank the reviewer for these considerations. As described in lines 271 to 274 of our original manuscript: “The first report contains Pillar 1 testing information spanning weeks 5 (2020) to 39 (2020), and Pillar 2 positivity ratios from week 19 up to week 39 (2020). The second report presents both Pillar 1 and Pillar 2 testing data from week 27 to week 44 (2020)”. Given the absence of Pillar 2 data early in the pandemic, we unfortunately cannot perform simulations using Pillar 2 data only. We should mention that we did consider using the REACT or ONS data suggested by the reviewer but found them to be inappropriate for this analysis. The first surveys were conducted in late April/early May 2020, and hence could not inform the early phase of the epidemic stage function. Furthermore, they suffer from expected sampling biases, as evidenced by discrepancies in coverage across regions and overall over-representation of white ethnicity respondents in the REACT study^[2]^ and regional sampling adjustments over time in the ONS data^[3]^.

Fortunately, since our original submission, the GOV.UK COVID-19 dashboard published the aggregate daily PCR tests and positivity data for seven regions of England. These are the most complete data of PCR positivity, which include household survey data. In the revised manuscript, we replace the Pillar 1 and Pillar 2 average results taken from the Public Health England weekly reports with the new datasets and re-run our model for the time-varying infection fatality ratios model. Since the shape of the PCR positivity ratios from both sources are nearly identical (**Figure 11** below), all results are unchanged.

**Figure 11. Comparison of shapes of PCR positivity from two different sources.** The red line shows the PCR test positivity ratios from the UK GOV dashboard while the green line shows the Pillar 1 and Pillar 2 average positivity ratio from the Public Health England weekly report.

1.8. ***I am not sure if there are any studies on age-specific antibody waning rates, but did the authors consider including location-specific seroreversion rate parameters? Given the argument for location-specific IFRs being driven by different age distributions, the same might be true of seroreversion rates. If identifiability is a concern, then the authors could use a multi-level model (ie. beta_i ~ Normal(beta_mean, var_beta))***

**Author response:** We did not find any evidence in the literature suggesting age dependence on antibody wanning. In all likelihood, seroreversion is a function of the intensity (maximum antibody titer) and quality (half-life of memory cells) of someone’s immune response to the virus. Several studies show that the amount of IgG antibody is correlated with the severity of infected individuals^[4],^^[5]^, which could implicate age structure as a driver of IFR differences across settings, given age is by far the most significant covariate in determining disease risk. However, longitudinal studies of the dynamics of SARS-CoV-2 neutralizing antibody responses found a marked variation from patient to patient, with age explaining only a small portion of the variance^[6]^. Whilst we think this is a good idea, we believe there is too much uncertainty in the evidence around it and will address it in future work if better data comes to light.

1.9. ***Further, the seroreversion rate is found to be higher here than in the Imperial report. Could the authors speculate on why this is?***

**Author response:** In fact, our mean estimate for the seroreversion rate is very similar to the one provided in the Imperial report (**Figure 12**). We believe this robustness is one of the main strengths of our approach – despite the different assumptions about the seroreversion rate that underpin our study and the methodology in the Imperial report, we obtain comparable mean estimates but our approach accounts for the different typologies of seroreversion observed in longitudinal studies^6^. There is mounting evidence from long term follow-up of convalescent patients that antibodies are detectable up to one year^[7],^^[8],^^[9],^^[10]^. Taking the Weibull curve in the Imperial report, 99.19% of seropositive individuals would serorevert by day 200, which seems to be at odds with the data. Our method employs an exponential distribution with a longer tail, resulting in 63.65% (95% Crl: (68.02%, 71.64%)) of seropositive individuals seroreverting in 200 days.

Notably, the estimation of the seroreversion rate can be biased by the antibody testing assay used to generate the seropositivity data the model is fit to. Whilst the Imperial report evaluated the distribution of seroreversion rate for the Abbott IgG assay, our analyses are based on serological data generated using the Euroimmun® assay. As mentioned in the Imperial report, “Abbott IgG antibody testing assay showed the most striking decline in sensitivity over time compared to other serological assays” which might explain their estimates for the Weibull function parameters.

**Figure 12. Probability of seropositivity persistence after seroconversion.** The green curve shows the probability curve from Imperial report^4^ and the orange curve and ribbon show the median and 95% Crl generated by our method when assuming a constant IFR.

One recent study from Public Heath England using Euroimmun® assay^[11]^ predicted that “proportions sero-reverting after 52 weeks were 59% (95% credible interval 50-68%)” while another study^[12]^ showed “after 21 weeks’ follow-up, 21.7% anti-S1 Euroimmun® assay measurements reverted to negative” which are more aligned with the exponential decay assumed in our analyses.

1.10. ***Please clarify the antigen target of the assays referred to (eg. Euroimmun® -> spike).***

**Author response:** We thank the reviewer for bring this up and have added this information to the revised manuscript.

1.11. ***Was there much correlation between the posteriors for IFRs and the seroreversion rate? If so, this would be very useful to report (possibly with 2-dimesional posterior density plots).***

**Author response:** We did not report this initially as we found there was not much correlation between the posteriors for IFRs and the seroreversion rate (**Figure 13**). Given this and other reviewer’s comments on the specifics around the IFR estimates reported, we must also stress that the main focus of this manuscript is to provide a robust method to infer cumulative exposure by triangulating seroprevalence data with mortality and test positivity data. In doing so, we provide an estimate of IFR, as have many other papers in the literature. However, the IFR estimate is intimately correlated with the accuracy of the mortality data as mentioned in the reply to **comment 1.4**. Regardless of what the true IFR is, our analyses indicate that, due to seroreversion, seroprevalence data will always under-estimate true exposure.

**Figure 13. Pairwise joint posterior distributions.** Beta is the seroreveriosn rate and gamma values give the IFRs for each region.

1.12. ***I may have missed something, but I really cannot see how eta and gamma can be estimated separately based on Equation 6. They are both just constants bounded between 0 and 1. Are the equations in this section correct, or have I just missed something obvious? Should it be alpha_i(t) = gamma_i*(1-eta_i * ES_i(t))? As with the seroreversion rate, if these parameters are correlated that would be useful to show.***

**Author response:** We thank the reviewer for noticing this. It should be alpha_i(t) = gamma_i*(1-eta_i * ES_i(t)). We corrected Equation (6) in the revised manuscript.

1.13. ***L150: I am not sure I follow that seroreversion on average 6 months post seroconversion *explains* a 0.17 odds ratio of reinfection within 6 months. It is certainly a related finding, but is the implication a) that reinfections did occur because of rapid seroreversion or b) reinfections were rare because seroreversion mostly happens after 6 months (and isn’t the referenced study 5 months)? This could be made clearer.***

**Author response:** We appreciate that this rationale could have been better laid out. Seropositivity is essentially a binary state that identifies whether a person has antibodies over a certain titer. Although there is no perfect corollary for immune protection, detectable antibody titers are quite protective against infection and disease. Thus, the transition between a seropositive state to a seronegative state can have implication in terms of the likelihood of reinfection. However, we now have a lot more evidence coming from longitudinal studies to inform the dynamics of antibody persistence as mentioned in the reply to **comment 1.9**. We have revised the manuscript to provide a better empirical support to our seroreversion rate estimates.

1.14. ***L155: I don’t think the relevance of serological data in a broad sense is called into question. Certainly, how these data are interpreted should be, but the data are still incredibly useful!***

**Author response:** Our claim was that serological data should not, on its own, inform policy since they are an imperfect measure of past exposure. We believe this is a valid point to make given the potential misuses of this type of data. We have made some modifications to the text to clarify this.

1.15. ***Figure 2: this is a lovely figure, but I do wonder if the data from Fig S3A should be included here. Those data are part of the fitting procedure, so it is a little misleading to show the fits just to seroprevalence. For example, how does the model estimate an uptick in exposures/seroprevalence in November in the North East and North West? This seems like magic based on the seroprevalence data alone. Furthermore, what was the rationale behind showing the map as of 17th October or just arbitrary (changes to the tier system)?***

**Author response:** Thanks for bringing this to our attention. The “magic” for estimating an uptick in exposure comes from the core principle of our approach – that cumulative mortality can inform the shape of past exposure (**Figure 14**). We chose to only display the seroprevalence data, as that is the only dataset the model is fit to. We do appreciate, however, that the mortality curves might aid interpretation and now present them in a supplementary figure. We also make it clearer in the text that predicted seroprevalence reflects the net effect of increases due to recent transmission (informed by lagged mortality) and decreases due to antibody waning (informed by the seroreversion rate).

**Figure 14. Cumulative deaths in seven regions of England.**

Furthermore, we only extend our window of exposure predict until October because serology data was not available after that date and, perhaps more importantly, IFR trends are expected to be disrupted with the introduction of new (potentially more virulent) strains and vaccines.

1.16. ***L162: I hate to be that person, but these are credible intervals, not confidence intervals.***

**Author response:** We thank the reviewer for bringing this to our attention. We have made the appropriate changes.

1.17. ***L217: I love this tactful start to the discussion.***

**Author response:** We thank the reviewer for their comment.

1.18. ***L290: It might be clearer to use (t-d_e) for the infections here.***

**Author response:** Thanks for bringing this to our attention. We agree and have modified this in the revised manuscript line 336, page 17.

1.19. ***L309: Please give the specificity and sensitivity of the assay.***

**Author response:** We have corrected this in the manuscript – line 315, page 16.

1.20. ***Eq1: Should it be beta * X_i (t)?***

**Author response:** Thanks for noticing this typo. We have made the necessary corrections.

1.21. ***L335: I’m not sure how this is a hierarchical model – you just present the likelihood and priors. If you had gamma_i ~ Normal(gamma, sigma) then that would make sense, but all of the parameters are independent.***

**Author response:** We agree that this is not technically a hierarchical model and have changed the text to reflect that.

1.22. ***L345: was there any rationale behind setting phi to 100, or just arbitrary?***

**Author response:** Thanks for bringing this to our attention. We set phi to 100 because it is consistent with a related study^[13]^, which was also looking at case counts. Also, the assumed large overdispersion errs on the side of caution and tries to account for the large uncertainty in the serological data.

2. **Reviewer 2**
   2.1. ***The study provides estimates of seroreversion, IFR and population exposure levels. The results are nicely presented. The methods seem sound and well-justified for this reviewer. It is difficult to place the results in terms of known reports of cases and deaths (and case fatality ratio). It would be beneficial to discuss the connection of these results to case and death data (and CFR) for clear interpretation.***

**Author response:** We appreciate the reviewer’s positive feedback. In the initial submission we presented a supplementary figure with the relevant epidemiological metrics in England over the course of the pandemic, including HFR and CFR. We now expand a bit on that to resolve any misunderstandings on why we chose test positivity over time as a proxy for epidemic stage progression and why we discarded all reported case data (**Figure 15**).

**Figure 15. Relevant epidemiological metrics in England over the course of the pandemic.** Case correction was done by taking the number of daily tests done on May 1^st^ and extrapolating the number of daily cases that would be reported if the testing effort had been constant over time, i.e., how many daily cases would be reported if 20,000 tests has been done every day. Note the different scale for mortality data used on panels B and C. In panel C we present the absolute number of deaths reported per day as a means of comparing its scale to the reported case data. In panel B we modify the mortality incidence scale to more easily compared its shape over time against that of the daily corrected cases and test positivity ratio curves.

From the figure above, it should be clear that case reporting (and consequently Case Fatality Ratio (CFR)) are highly influenced by a hyper volatile testing effort (number of people tested per day). This causes a disconnect between the shape of mortality incidence and test positivity curves on one end and the case incidence curve at the other end. More worryingly, the reported case curve peaks at roughly the same time as the mortality data (early April) which is clearly biologically implausible. The expected delay between case incidence peak and mortality incidence peak is retrieved after applying the testing effort correction to the case data (panel C). Although Hospitalization Fatality Ratio (HFR) seems to follow the expected dynamics closer than CFR, we would still argue that test positivity ratio over time (and testing effort corrected case data) are the best indicators of the true shape of transmission intensity over time, along with mortality data (panel B).

3. ***Reviewer 3***
   3.1. ***What are the main claims of the paper and how significant are they for the discipline?***

***The work brings up the challenge to truly estimate the proportion of the population exposed to SARS-COV-2, the severity of the disease determined by the IFR and the seroreversion time, a limit marker for detecting antibodies in an infected individual. The authors estimate seroreversion time around six months, infection fatality rate varying from 0.0049 to 0.0118, and variations of the population exposed to SARS-COV-2 from 5% to 20% in seven regions in England as of October 2020. Their mathematical modelling relied on data of observed daily mortality, SARS-CoV-2 antibody seroprevalence measurements, and weekly positivity ratios of laboratory-confirmed COVID-19 cases aggregated for each of the regions studied. The results obtained can significantly help in the pandemic and guide the application of non-pharmaceutical and vaccinations strategies.***

**Author response:** We appreciate the reviewer’s positive feedback and are very happy to respond to their comments.

3.2. ***However, the authors change the focus of the work objective in the Result and Discussion sections and bring up a hypothetical analysis and discussions about the frailty selection process. Their method and data do not represent biological, social or economic factors, which influence the concept of frailty, which is instead an unobserved susceptibility to adverse outcomes. Thus, the paper must be not accepted for publication unless major corrections are performed.***

**Author response:** We thank the reviewer for these observations. We agree that we need to adjust the topics in the discussion section to the relevant material addressed in the manuscript. Please also see our detailed responses to **comments 3.14** and **3.19** in this document.

3.3. ***Are these claims novel? If not, which published articles weaken the claims of originality of this one?***

***The methodology and results concerning the estimation of the population exposure, fatality rate and time of seroreversion are very similar to the work of Shioda, Kayoko, et al. "Estimating the cumulative incidence of SARS-CoV-2 infection and the infection fatality ratio in light of waning antibodies." medRxiv (2020), https://doi.org/10.1101/2020.11.13.20231266, posted on November 16, 2020. Despite this, the analysis performed by the draft submitted by Aguas still has originality and is adapted to a different setting.***

**Author response:** We appreciate the reviewer’s positive feedback about our manuscript’s originality.

3.4. ***Are the claims properly placed in the context of the previous literature? Have the authors treated the literature fairly?***

***No. In some parts of the text, the authors lack literature review to support their claims. Please, see the General Comments below.***

**Author response:** We thank the reviewer for calling our attention to this issue and have made an effort to add more references supporting the claims in the text. Please also see the detailed responses to **comments 3.14** and **3.21**.

3.5. ***The authors do not mention the findings regarding the estimated fatality rate in the abstract.***

**Author response:** We thank the reviewer for their suggestion. We have added the estimated IFR range across regions in England to the abstract.

3.6. ***In the manuscript, the authors mention that the “REACT” study had estimated a 13% exposure proportion in London, from June 20th to July 13th 2020. How do these values compare to estimates made by the authors for a more extended period up to November 7th? What is the estimated exposure and IFR in the literature for the other regions compared to the results obtained in Figure 2? for which period?***

**Author response:** We believe that the reviewer misunderstood the information we extracted from the REACT study and hope the statement below will clarify this.

In the original manuscript, we mentioned that “*seroprevalence in London was reported by REACT to be 13.0% (12.3–13.6) for the period 20 June to 13 July 2020. In comparison, the London blood donor time series indicated seroprevalence to be 13.3% (8.4–16) on 21 June 2020.*” The REACT study reported a seroprevalence instead of “exposure” to be 13.0% for the period 20 June to 13 July 2020; therefore, it should not be compared with our estimations of exposure for the same period. The reason why we mention the REACT study is that we think the dataset of England blood donors used in our paper is representative and informative because it “*informed the national COVID-19 serological surveillance and its data collection was synchronous with the REACT study*”, see lines 106-107 in the original manuscript.

3.7. ***The authors claim that reducing the ratio of deaths to infections is due to frailty (lines 175-180); however, they do not consider the seasonality of respiratory diseases, which usually has a lower prevalence in hot seasons. Also, improvement of healthcare managements and patients treatments can be an issue for a reduction in IFR. The work lacks discussion about that.***

**Author response:** There seems to be some confusion around what the frailty hypothesis is trying to explain. From the time varying IFR estimates we observe two interesting trends: (1) spatial heterogeneity in baseline (first wave) IFRs; (2) faster IFR decline over time in London compared to other regions. Although we agree other factors can be involved, we believe the frailty hypothesis is the most parsimonious one to explain (2). We have made this clearer in the main text and add supplementary analyses addressing (1) and (2). Please refer to **comment 1.3** for more details.

3.8. ***Figure 2 and 3 are repeated in the manuscript.***

**Author response:** Figure 2 shows the “Time course of the SARS-CoV-2 pandemic up to 7 November 2020 for seven regions in England **for the constant IFR model**”. Figure 3 shows “Time course of the SARS-Cov-2 pandemic up to 7 November 2020 for seven regions in England **for the time varying IFR model**”. This distinction has been made clearer in the figure titles.

3.9. ***A delicate issue in their analysis is the assumption of uninformative beta priors for the parameters estimated. Uninformative priors are vague and may affect the posterior distribution of the results.***

**Author response:** We thank the reviewer for these observations. Uninformative priors make the posterior distributions of parameters largely depend on the data^[14]^. There are no obvious prior distributions choices for regional IFRs and seroreversion ratios, therefore, we think it is reasonable to use uninformative priors to reflect our insufficient knowledge regarding those parameters. To address this issue further, we replicated our results assuming a Weibull distribution with shape parameter as 3.67 and scale parameter as 130.4^[15]^ to inform beta. We found the posterior estimation of the seroreversion rate and IFRs are very robust to the choice of priors for both constant IFR and time varying IFR models (**see Tables S4 and S5 in the revised manuscript**).

3.10. ***Also, in the literature, it is shown that the time from symptom onset to seroconversion is consistent with the Weibull distribution. It must be taken into account in the analysis performed.***

**Author response:** We thank the reviewer for these observations. We have clarified that the choice of a fixed delay is a simplification of the delay-distribution approaches used elsewhere in the revised manuscript. Additionally, we performed a sensitivity analysis on the assumptions of fixed delays between exposure and seroconversion and between a PCR test and seroconversion. Please refer to our response to **comment 1.6** above.

3.11. ***The applicability of the proposed methods to other places worldwide can be influenced by the serological assays methodologies, which affect, for instance, the effects of variations on the time lag between exposure and seroconversion. How would it affect the analysis performed? How much does it influence the measures obtained in the results?***

**Author response:** We thank the reviewer for these observations. This and several of the points raised earlier lead us to perform an exhaustive sensitivity analysis that does include an exploration of the robustness of our original results to assumptions in the time lag between exposure and seroconversion. A summary of the sensitivity analysis can be found in the supplementary methods and Tables S4 and S5. Please see the response to **comment 3.12** where we discuss context dependencies and the range of applicability of our methodology.

3.12. ***What are the recommendations and limitations of the applicability of the work worldwide?***

**Author response:** We thank the reviewer for this suggestion and agree that “the applicability of the proposed methods to other places worldwide” is really a good discussion point. We have added a section to the discussion as per below:

“We should mention some details that potentially limit the applicability of the methodology presented here to other countries, especially low- and middle-income countries (LMICs). The most pertinent is one of data quality. Whereas our assumption that COVID-19 deaths are nearly perfectly reported in England is a plausible one, it is very unlikely to hold for other countries across the globe. To account for potential under-reporting, we could include a constant or time-varying reporting ratio to transform reported deaths into “likely” deaths. The direct consequence of using predicted deaths as a model input would be that any IFR estimates would be very difficult to disentangle from the underlying reporting ratio. The quality of the seroprevalence data itself is paramount and the data collection protocol can severely influence the obtained estimates as evidence by two concurrent seroprevalence studies in Manaus, Brazil. Whereas one reports a raw seroprevalence of ~ 40% using the Abbot test, the other reports ~13% antibody positivity using the WONDFO SARS-CoV-2 antibody test, both measured in May 2020. A significant difference is that the first study used blood donor samples, whereas the second relates to household surveys. Another issue that is likely to be relevant to many LMICS, is that the provision of a reliable level of uncertain around the seroreversion estimate relies on having several sequential seroprevalence measurements. In countries with a very limited capacity for doing serosurveys, we suggest using the posterior distribution for seroreversion provided here as an informative prior and proceed to estimate the infection fatality ratios and exposure profiles. However, we should not that the thresholds of seropositive and seronegative assignment vary across the assays, hindering the applicability of estimates resulting from data generated with a specific assay to other setting where different assays might be used.”

3.13. ***Additionally, the discussion section is poorly written and does not compare the work with others performed on the literature. For instance, the author mention: “The associated estimate of time to seroreversion of 176 days (95% CrI: 159-197) lies within realistic limits derived from independent sources.” Which sources (references)? “The total exposure in regions of England estimated using this method is more than double the last seroprevalence measurements.” In which references?***

**Author response:** We appreciate that for the sake of clarity, we could have repeated the referencing here, but we provide all of these in the results section when these figures were presented. We have revised the discussion to include references to the most recent data and IFR estimates in the literature.

4. ***Reviewer 4***
   4.1. ***As I understand from the authors' modeling analyses of the English seroprevalence data and the conclusion arrived at, the time-invariant IFR model (results in Figure 2) is as good as the time-dependent IFR model (results in Figure 3). This is a good news from the model parsimony point-of-view - a simple model performs as good as a slightly more complex one does. However, I do not see a connection between this ("no statistically significant time-dependence on IFR was inferred") and the authors' point on line 197 "... suggesting this phenomenon is dependent on age structure." The authors have not performed their modeling analyses using an age-structured model. Therefore, the authors need to observe some caution before arriving at what they said on the line. As I think, it needs a little bit more unpacking. Or modification in the statement.***

**Author response:** We thank the reviewer for their positive comments. We have redrafted the results and discussions section to address the two main trends in our time varying IFR estimates: (1) spatial heterogeneity in baseline (first wave) IFRs; (2) faster IFR decline over time in London compared to other regions. We also present a new series of data explorations (see reply to **comment 1.3** for more detail) that offer support to our hypothesis on how to explain both (1) and (2).

4.2. ***The data presented in Figures 2 and 3 have calendar months as independent variable. But the authors describe the data in the units of weeks of 2020 (lines 270 to 273). A reader like myself starts wondering which week number corresponds to which calendar month.***

**Author response:** Thank you for calling our attention to the disconnect. The data description mentioned the week number since it was an identifier of the report used – they are named according to week number. However, we have since found a more cohesive database and have changed the data description accordingly.

4.3. ***The authors have made a simplifying assumption in their models, "From the moment of exposure, individuals seroconvert a fixed 21 days later ..." I was expecting to see some sensitivity analysis around this assumption of a fixed 21-day period: how will the [main] result change if this fixed time-period is shortened or lengthened by a week?***

**Author response:** We thank the reviewer for the comment. Please refer to our reply to **comment 1.6** above.

1. https://www.nomisweb.co.uk/datasets/pestsyoala
2. https://www.thelancet.com/journals/lanepe/article/PIIS2666-7762(21)00075-2/fulltext
3. https://www.ons.gov.uk/peoplepopulationandcommunity/healthandsocialcare/conditionsanddiseases/methodologies/covid19infectionsurveypilotmethodsandfurtherinformation
4. https://www.nature.com/articles/s41564-020-00813-8/figures/5
5. https://www.sciencedirect.com/science/article/pii/S0009898120302631
6. https://www.thelancet.com/journals/lanmic/article/PIIS2666-5247(21)00025-2/fulltext
7. https://www.medrxiv.org/content/10.1101/2021.04.19.21255720v3
8. https://www.thelancet.com/journals/laninf/article/PIIS1473-3099(20)30943-9/fulltext
9. https://academic.oup.com/cid/advance-article/doi/10.1093/cid/ciab172/6149064
10. https://www.ncbi.nlm.nih.gov/pmc/articles/PMC7816882/
11. Harris, Ross J., et al. "Serological surveillance of SARS-CoV-2: Six-month trends and antibody response in a cohort of public health workers." Journal of Infection 82.5 (2021): 162-169.
12. Manisty, Charlotte, et al. "Time series analysis and mechanistic modelling of heterogeneity and sero-reversion in antibody responses to mild SARS‑CoV-2 infection." EBioMedicine 65 (2021): 103259.
13. https://elifesciences.org/articles/58785
14. Winkler, Robert L. "The assessment of prior distributions in Bayesian analysis." Journal of the American Statistical association 62.319 (1967): 776-800.
15. Imperial report 34
