# Supplementary material for: Levels of SARS-CoV-2 population exposure are considerably higher than suggested by seroprevalence surveys
Source: PLoS Comput Biol. 2021 Sep 20;17(9):e1009436. doi: 10.1371/journal.pcbi.1009436 (PMC8483393; doi:10.1371/journal.pcbi.1009436)
Supplement: Attachment — Submitted filename: Reply to reviewers_r2.docx [file pcbi.1009436.s025.docx]

**Reply to reviewers’ comments**, **submission PCOMPBIOL-D-21-00584**:

"Levels of SARS-CoV-2 population exposure are considerably higher than suggested by seroprevalence surveys"

1. **Reviewer 1**
   1.1. ***My initial comments on this manuscript were very positive, recommending minor revisions to explore the impact of some key, fixed assumptions (namely the death data and reporting delays) on the estimated seroreversion rate and IFR. The authors have responded very thoroughly and added some clear sensitivity analyses to address these concerns. The arguments against some of my suggestions are convincing (e.g., not including a death reporting rate parameter). I also think the extended discussion describing the frailty hypothesis more clearly is a great addition. Overall, I think the authors have done a fantastic job revising the manuscript and recommend publication.***

**Author response:** We truly appreciate the reviewer’s prior feedback and firmly believe their contribution led to significant improvements to the paper’s potential reach and impact.

1.2. ***I would mention in the supplementary figure captions showing the different scenarios that the scenario assumptions are shown in Table S4. Same for eg. the legend in Figure S11. I would just try to make it clearer which numbers refer to which assumptions and be consistent with terms like “Model 2” vs. “Scenario 2”.***

**Author response:** We agree that referring to Table S4 might improve readability. We have made the suggested changes and made sure consistency was maintained when mentioning the models used.

1.3. ***Regarding the regression analysis explaining region-specific differences in IFRs: there seems to be a slight discrepancy between the response document and Figure S4. The response document states that % of people over 45 explains 61% of the variance, whereas the figure shows proportion over 60 as the cutoff. The main text also refers to the proportion of people in care homes explaining 75% of variance, not the figure for people over 45.***

**Author response:** We have made numerous regressions, too many to include in either the response or the paper. We felt that if we were to include a single age breakpoint in the manuscript, 60 years of age (or perhaps 65) would probably make more sense to a wider audience, particularly since we present it alongside a regression against the proportion of people in care homes. However, since in the text we do reference 45 years of age as a breakpoint above which disease and mortality risk start to increase significantly, we have reverted to plotting that variable in the figure.

1.4. ***Related to the new regression analyses: the description of what was done is too brief, and the regression coefficients and CIs should be reported. It is also not quite clear if the authors fit a single regression including all of the demographic covariates, or if they fit separate simple linear regressions to each. I actually think I could be missing a file: the authors refer to supplementary methods, but I cannot find them anywhere in the system. Please just check these are included and that the regression methods/results are described in detail. Same goes for the delta_p/delta_e analyses.***

**Author response:** For simplicity, we include all relevant information on the regression and sensitivity analyses in the Methods section of the paper.

1.5. ***Response 1.21: the methods still refer to this as a hierarchical model (L427).***

**Author response:** We did notice this issue before receiving the reviewer’s comments and would have corrected it regardless. We had some issues with manuscript version control with several authors making changes to the text using different text editors and OS versions. Apologies for the confusion.

1.6. ***L38 in author summary: typo “take thin into account”. Inconsistent use of “” and ‘’ eg. L127/128.***

**Author response:** We have made the appropriate corrections.

2. **Reviewer 4**
   2.1. ***I congratulate the authors for revising their original manuscript as per the comments/suggestions made by all reviewers. However, I realized that the authors might have forgotten to run spelling checker or a final proof-reading - some words need to be corrected. For example, in line 38 thin -> this?***

**Author response:** We appreciate the reviewer’s feedback. As mentioned in **1.5** above, we had some issues with manuscript version control with several authors making changes to the text using different text editors and OS versions. We have ensured the revised version is appropriately proofread.

2.2. ***In addition, I see that the authors still use the word "hierarchical" in line 427 of the revised submission. They had responded to Comment 1.21 saying that [and I quote then here, "We agree that this is not technically a hierarchical model and have changed the text to reflect that."] that they would drop this word. So, what happened? I ask authors to be mindful of following things in their revision as per their own responses.***

**Author response:** Please refer to point **1.5** above.
